# Supplementary material for: Polyubiquitin architecture editing on collided ribosomes maintains persistent RQC activity
Source: EMBO J. 2025 Sep 16;44(21):6051–77. doi: 10.1038/s44318-025-00568-0 (PMC12583759; doi:10.1038/s44318-025-00568-0)
Supplement: Supplementary file 1 — Appendix [file 44318_2025_568_MOESM1_ESM.pdf]

Appendix for
Editing of the polyubiquitin architecture on the collided ribosome maintains persistent RQC activity

Table of Contents

Appendix Tables
Appendix Table S1. pp. 2-6
Appendix Table S2. pp. 7-8

Appendix Table S1

Yeast strains used in study

**strain name**	***Feature***	**Source**
YKI1587	*uS10Δ::natMX4 p414uS10p-uS10-CYCt*	This study
YST209	*uS10Δ::natMX4 p414uS10p-uS10-3HA-CYCt*	This study
YST094	*uS10Δ::natMX4 p414uS10p-uS10-3HA-6His-CYCt*	This study
YST285	*uS10Δ::natMX4 p414uS10p-yUb G76V-uS10 K8R 8-121-3HA-6His-CYCt*	This study
	BY4741 WT	Lab. Stock
	BY4741 ubp1Δ::KAN	Lab. Stock
	BY4741 ubp2Δ::KAN	Lab. Stock
	BY4741 ubp3Δ::KAN	Lab. Stock
	BY4741 ubp4Δ::KAN	Lab. Stock
	BY4741 ubp5Δ::KAN	Lab. Stock
	BY4741 ubp6Δ::KAN	Lab. Stock
	BY4741 ubp7Δ::KAN	Lab. Stock
	BY4741 ubp8Δ::KAN	Lab. Stock
	BY4741 ubp9Δ::KAN	Lab. Stock
	BY4741 ubp11Δ::KAN	Lab. Stock
	BY4741 ubp12Δ::KAN	Lab. Stock
	BY4741 ubp13Δ::KAN	Lab. Stock
	BY4741 ubp14Δ::KAN	Lab. Stock
	BY4741 ubp15Δ::KAN	Lab. Stock
	BY4741 ubp16Δ::KAN	Lab. Stock
	BY4741 otu1Δ::KAN	Lab. Stock
	BY4741 otu2Δ::KAN	Lab. Stock
	BY4741 yuh1Δ::KAN	Lab. Stock
YST210	*uS10Δ::natMX4 p414uS10p-uS10K6/8R-3HA-CYCt*	This study
YST213	*uS10Δ::natMX4 ubp2Δ::kanMX4 p414uS10p-uS10-3HA-CYCt*	This study
YST214	*uS10Δ::natMX4 ubp2Δ::kanMX4 p414uS10p-uS10K6/8R-3HA-CYCt*	This study
YST238	*uS10Δ::natMX4 ubp3Δ::kanMX4 p414uS10p-uS10-3HA-CYCt*	This study
YST239	*uS10Δ::natMX4 ubp3Δ::kanMX4 p414uS10p-uS10K6/8R-3HA-CYCt*	This study

	W303-1a	Lab. Stock, Parent
YST034	*ubp2Δ::kanMX4*	This study
YKI1058	*ubp3Δ::hygMX4*	This study
YST045	*ubp2Δ:kanMX4 ubp3Δ:hygMX4*	This study
YKK347	*ltn1Δ::kanMX4*	Matsuda *et al.*, 2014
YST029	*ltn1Δ::natMX4 ubp2Δ::kanMX4*	This study
YAI027	*ltn1Δ:: kanMX4 ubp3Δ::hyMX4*	This study
YST056	*ubp2Δ::kanMX4 ubp3Δ::hygMX4 ltn1Δ::natMX4*	This study
YKK110	*hel2Δ::natMX4*	Matsuo *et al.*, 2017
YKI765	*hel2Δ::natMX4 ltn1Δ::kanMX4*	Matsuo *et al.*, 2017
YST003	*hel2Δ::natMX4 ubp2Δ::hygMX4*	This study
YST054	*ubp2Δ::hygMX4 hel2Δ::natMX4 ltn1Δ:kanMX4*	This study
YAI026	*ubp3Δ::hygMX4 hel2Δ::natMX4*	This study
YST055	*ubp3Δ::hygMX4 hel2Δ::natMX4 ltn1Δ::kanMX4*	This study
YSS2191	*MATa lys2-801 leu2-3,2-112 ura3-52 his3-Δ200 trp1-1 ubi1-Δ1::TRP1 ubi2-Δ2::ura3 ubi3-Δub-2 ubi4Δ2::LEU2 (YEpKan-TEF1p-kozak-yUb-CYC1t) (pUB100)*	This study
YSS2192	*MATa lys2-801 leu2-3,2-112 ura3-52 his3-Δ200 trp1-1 ubi1-Δ1::TRP1 ubi2-Δ2::ura3 ubi3-Δub-2 ubi4Δ2::LEU2 (YepKan-TEF1p-kozak-yUbK63R-CYC1t) (pUB100)*	This study
YST059	*YSS2191 ubp2Δ::natMX4*	This study
YST060	*YSS2192 ubp2Δ::natMX4*	This study
YST061	*YSS2191 ubp3Δ::natMX4*	This study
YST062	*YSS2192 ubp3Δ::natMX4*	This study
YST032	*ubp2-3HA::HIS3:HisMX6*	This study
Y45	*ubp3-3HA::HIS3:HisMX6*	This study
YST211	*uS10Δ::natMX4 p414uS10p-uS10K6R-3HA-CYCt*	This study
YST212	*uS10Δ::natMX4 p414uS10p-uS10K8R-3HA-CYCt*	This study
YST101	*uS10Δ::natMX4 ubp2Δ::kanMX4 p414uS10p-uS10-3HA-6His-CYCt*	This study
YST102	*uS10Δ::natMX4 ubp3Δ::kanMX4 p414uS10p-uS10-3HA-6His-CYCt*	This study

YST095	*uS10Δ::natMX4 p414uS10p-uS10K6/8R-3HA-6His-CYCt*	This study
YST143	*MATa lys2-801 leu2-3,2-112 ura3-52 his3-Δ200 trp1-1 ubi1-Δ1::TRP1 ubi2-Δ2::ura3 ubi3-Δub-2 ubi4Δ2::LEU2 (YEpKan-TEF1p-kozak-yUbR54A-CYC1t) uS10Δ::natMX4 p416uS10p-uS10-3HA-6His-CYCt*	This study
YST188	*MATa lys2-801 leu2-3,2-112 ura3-52 his3-Δ200 trp1-1 ubi1-Δ1::TRP1 ubi2-Δ2::ura3 ubi3-Δub-2 ubi4Δ2::LEU2 (YEpKan-TEF1p-kozak-yUbR54A-CYC1t) uS10Δ::natMX4 p416uS10p-uS10K6/8R-3HA-6His-CYCt*	This study
YST144	*YST143 ubp2Δ::kanMX4*	This study
YST235	*YST143 ubp3Δ::kanMX4*	This study
YST227	*uS10Δ::natMX4 p414uS10p-uS10K6R-3HA-6His-CYCt*	This study
YST228	*uS10Δ::natMX4 ubp2Δ::kanMX4 p414uS10p-uS10K6R-3HA-6His-CYCt*	This study
YST518	*uS10Δ::natMX4 ubp2Δ::hph ubp3Δ::kanMX4 p414uS10p-uS10-3HA-6His-CYCt*	This study
YST292	*uS10Δ::natMX4 ltn1Δ::kanMX4 p414uS10p-yUb G76V-uS10 K8R 8-121-3HA-6His-CYCt*	This study
YST293	*uS10Δ::natMX4 ltn1Δ::kanMX4 p414uS10p-yUb K48R G76V-uS10 K8R 8-121-3HA-6His-CYCt*	This study
YST294	*uS10Δ::natMX4 ltn1Δ::kanMX4 p414uS10p-yUb K63R G76V-uS10 K8R 8-121-3HA-6His-CYCt*	This study
YST295	*uS10Δ::natMX4 ltn1Δ::kanMX4 p414uS10p-yUb K48R K63R G76V-uS10 K8R 8-121-3HA-6His-CYCt*	This study
YST331	*uS10Δ::natMX4 ltn1Δ::kanMX4 p414uS10p-yUb K29R G76V-uS10 K8R 8-121-3HA-6His-CYCt*	This study
YST332	*uS10Δ::natMX4 ltn1Δ::kanMX4 p414uS10p-yUb K29R K48R G76V-uS10 K8R 8-121-3HA-6His-CYCt*	This study
YST333	*uS10Δ::natMX4 ltn1Δ::kanMX4 p414uS10p-yUb K29R K63R G76V-uS10 K8R 8-121-3HA-6His-CYCt*	This study
YST334	*uS10Δ::natMX4 ltn1Δ::kanMX4 p414uS10p-yUb K29R K48R K63R G76V-uS10 K8R 8-121-3HA-6His-CYCt*	This study
YST286	*uS10Δ::natMX4 p414uS10p-yUb K48R G76V-uS10 K8R 8-121-3HA-6His-CYCt*	This study
YST287	*uS10Δ::natMX4 p414uS10p-yUb K63R G76V-uS10 K8R 8-121-3HA-6His-CYCt*	This study
YST288	*uS10Δ::natMX4 p414uS10p-yUb K48R K63R G76V-uS10 K8R 8-121-3HA-6His-CYCt*	This study
YST327	*uS10Δ::natMX4 p414uS10p-yUb K29R G76V-uS10 K8R 8-121-3HA-6His-CYCt*	This study
YST328	*uS10Δ::natMX4 p414uS10p-yUb K29R K48R G76V-uS10 K8R 8-121-3HA-6His-CYCt*	This study

YST329	*uS10Δ::natMX4 p414uS10p-yUb K29R K63R G76V-uS10 K8R 8-121-3HA-6His-CYCt*	This study
YST330	*uS10Δ::natMX4 p414uS10p-yUb K29R K48R K63R G76V-uS10 K8R 8-121-3HA-6His-CYCt*	This study
YST300	*uS10Δ::natMX4 hel2Δ::kanMX4 p414uS10p-yUb G76V-uS10 K8R 8-121-3HA-6His-CYCt*	This study
YST301	*uS10Δ::natMX4 hel2Δ::kanMX4 p414uS10p-yUb K48R G76V-uS10 K8R 8-121-3HA-6His-CYCt*	This study
YST302	*uS10Δ::natMX4 hel2Δ::kanMX4 p414uS10p-yUb K63R G76V-uS10 K8R 8-121-3HA-6His-CYCt*	This study
YST303	*uS10Δ::natMX4 hel2Δ::kanMX4 p414uS10p-yUb K48R K63R G76V-uS10 K8R 8-121-3HA-6His-CYCt*	This study
YST352	*uS10Δ::natMX4 hel2Δ::kanMX4 p414uS10p-yUb K29R G76V-uS10 K8R 8-121-3HA-6His-CYCt*	This study
YST353	*uS10Δ::natMX4 hel2Δ::kanMX4 p414uS10p-yUb K29R K48R G76V-uS10 K8R 8-121-3HA-6His-CYCt*	This study
YST354	*uS10Δ::natMX4 hel2Δ::kanMX4 p414uS10p-yUb K29R K63R G76V-uS10 K8R 8-121-3HA-6His-CYCt*	This study
YST355	*uS10Δ::natMX4 hel2Δ::kanMX4 p414uS10p-yUb K29R K48R K63R G76V-uS10 K8R 8-121-3HA-6His-CYCt*	This study
YST337	*uS10Δ::natMX4 ufd4Δ::kanMX4 p414uS10p-yUb G76V-uS10 K8R 8-121-3HA-6His-CYCt*	This study
YST338	*uS10Δ::natMX4 ufd4Δ::kanMX4 p414uS10p-yUb K48R G76V-uS10 K8R 8-121-3HA-6His-CYCt*	This study
YST339	*uS10Δ::natMX4 ufd4Δ::kanMX4 p414uS10p-yUb K63R G76V-uS10 K8R 8-121-3HA-6His-CYCt*	This study
YST340	*uS10Δ::natMX4 ufd4Δ::kanMX4 p414uS10p-yUb K48R K63R G76V-uS10 K8R 8-121-3HA-6His-CYCt*	This study
YST341	*uS10Δ::natMX4 ufd4Δ::kanMX4 p414uS10p-yUb K29R G76V-uS10 K8R 8-121-3HA-6His-CYCt*	This study
YST342	*uS10Δ::natMX4 ufd4Δ::kanMX4 p414uS10p-yUb K29R K48R G76V-uS10 K8R 8-121-3HA-6His-CYCt*	This study
YST343	*uS10Δ::natMX4 ufd4Δ::kanMX4 p414uS10p-yUb K29R K63R G76V-uS10 K8R 8-121-3HA-6His-CYCt*	This study
YST344	*uS10Δ::natMX4 ufd4Δ::kanMX4 p414uS10p-yUb K29R K48R K63R G76V-uS10 K8R 8-121-3HA-6His-CYCt*	This study

YST234	*uS10D:natMX4 ufd4Δ::kanMX4 p414uS10p-uS10-3HA-6HIS*	This study
YST520	*uS10Δ::natMX4 ubp2Δ::hph ufd4Δ::kanMX4 p414uS10p-uS10-3HA-6HIS*	This study
YST203	*ufd4Δ:natMX4*	This study
YST378	*ufd4Δ:natMX4 ltn1Δ::K. lactis LEU2*	This study
YST531	*ski2Δ:kanMX4 uS10Δ:natMX4 p414uS10p-yUb K63 only G76V-uS10 K8R 8-121-3HA-CYCt*	This study
YST532	*ski2Δ:kanMX4 uS10Δ:natMX4 p414uS10p-yUb K63 only G76V-uS10 K8R 8-121-3HA-CYCt*	This study
YST543	*ski2Δ:kanMX4 uS10Δ:natMX4 hel2Δ:hph p414uS10p-yUb K48 only G76V-uS10 K8R 8-121-3HA-CYCt*	This study

Appendix Table S2

Plasmids used in study

**Plasmid name**	**Feature**	**Source**
pST001	*p415uS10p-uS10-3HA-CYCt*	This study
pLD003	*p416GPDp-GFP-R(CGN)12-FLAG-HIS3-CYCt*	Dimitrova *et al.*, 2009
pKI187	*p416GPDp-Hel2-FLAG-CYCt*	This study
pST014	*p416uS10p-uS10-3HA-CYCt*	This study
pST016	*p416uS10p-uS10K6R-3HA-CYCt*	This study
pST051	*p414uS10p-uS10-3HA-6xHis-CYCt*	This study
pST069	*p415GPDp-Hel2-V5-CYCt*	This study
pKI191	*p416GPDp-uL23-FLAG-CYCt*	This study
pKI236	*p414uS10p-uS10-3HA-CYCt*	Ikeuchi *et al.,* 2019
pKI237	*p414uS10p-uS10 K6R K8R-3HA-CYCt*	Ikeuchi *et al.,* 2019
pKI238	*p414uS10p-uS10 K6R-3HA-CYCt*	This study
pKI239	*p414uS10p-uS10 K8R-3HA-CYCt*	This study
pST081	*p416uS10p-uS10-3HA-6xHis-CYCt*	This study
pST097	*p413GPDp-Hel2-V5-CYCt*	This study
pST109	*pRS317GPDp-uL23-FLAG-CYCt*	This study
pST083	*p415GPDpUbp2-TEV-3xFLAG-CYC1t*	This study
pST045	*p415GPDp-ubp2(C745S)-TEV-3xFLAG-CYCt*	This study
pST006	*p415GPDp-ubp3-TEV-3×FLAG-CYCt*	This study
pST046	*p415GPDp-ubp3(C496A)-TEV-3xFLAG-CYCt*	This study
pST137	*p414uS10p-uS10 K6R -3HA-6xHis-CYCt*	This study
pST158	*p414uS10p-yUb G76V-uS10 K8R 8-121-3HA-6His-CYCt*	This study
pST159	*p414uS10p-yUb K29R G76V-uS10 K8R 8-121-3HA-6His-CYCt*	This study
pST160	*p414uS10p-yUb K48R G76V-uS10 K8R 8-121-3HA-6His-CYCt*	This study
pST161	*p414uS10p-yUb K63R G76V-uS10 K8R 8-121-3HA-6His-CYCt*	This study
pST162	*p414uS10p-yUb K29R K48R G76V-uS10 K8R 8-121-3HA-6His-CYCt*	This study
pST163	*p414uS10p-yUb K29R K63R G76V-uS10 K8R 8-121-3HA-6His-CYCt*	This study
pST164	*p414uS10p-yUb K48R K63R G76V-uS10 K8R 8-121-3HA-6His-CYCt*	This study
pST165	*p414uS10p-yUb K29R K48R K63R G76V-uS10 K8R 8-121-3HA-6His-*	This study

	CYCt	
pST320	*p414uS10p-yUb K63 only G76V-uS10 K8R 8-121-3HA-CYCt*	This study
pST321	*p414uS10p-yUb K48 only G76V-uS10 K8R 8-121-3HA-CYCt*	This study
p628	*p425GALp-Slh1-FTP*	Matsuo *et al.*, 2022
p542	*p424GALp-Cue3-Flag*	Matsuo *et al.*, 2023
p544	*p426GALp-Rqt4-Flag*	Matsuo *et al.*, 2023
