# Supplementary figures and images for: Polyubiquitin architecture editing on collided ribosomes maintains persistent RQC activity
Source: EMBO J. 2025 Sep 16;44(21):6051–77. doi: 10.1038/s44318-025-00568-0 (PMC12583759; doi:10.1038/s44318-025-00568-0)

Figure 1A

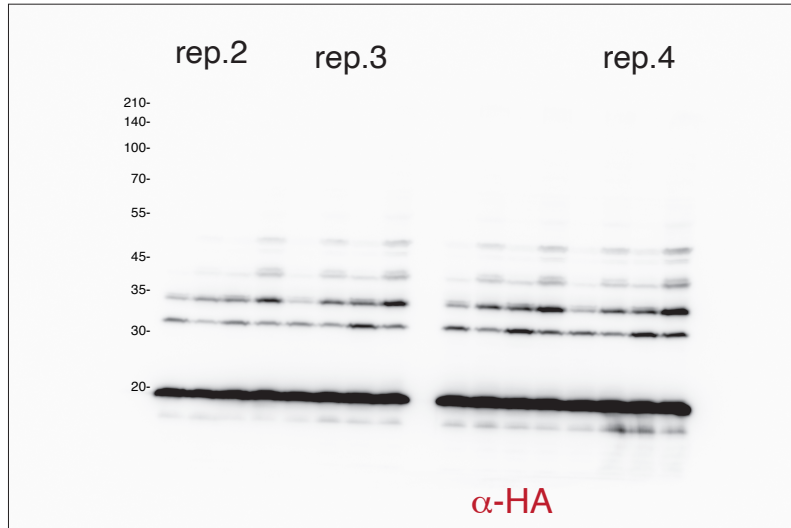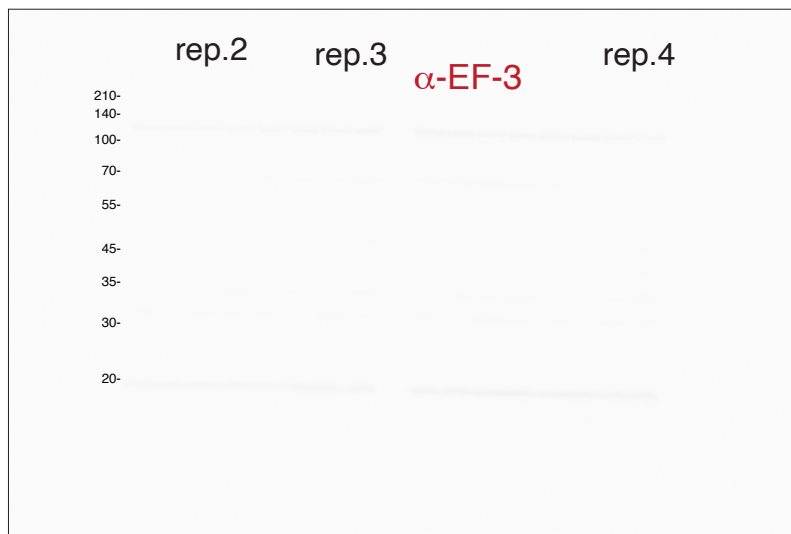

Supplement: Supplementary file 3 — Source data Fig. 1 [file 44318_2025_568_MOESM3_ESM.zip › Figure1/1A/Fig.1A_replicates.pdf]

Figure 1A

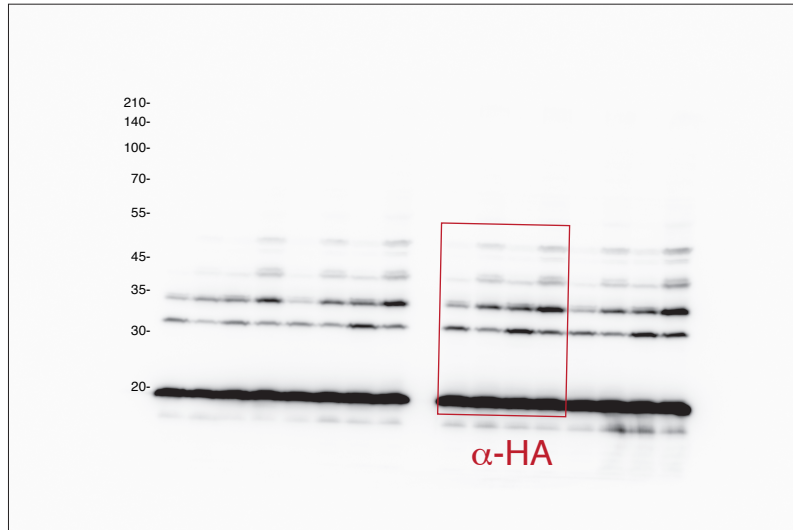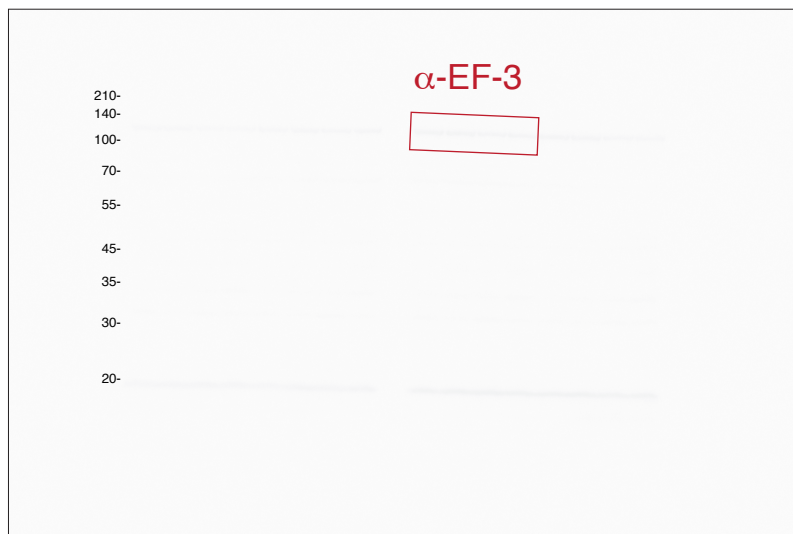

Supplement: Supplementary file 3 — Source data Fig. 1 [file 44318_2025_568_MOESM3_ESM.zip › Figure1/1A/Fig,1A.pdf]

Figure 1B

rep.2

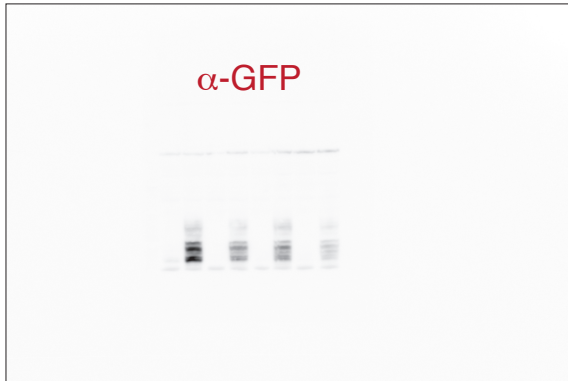

rep.3

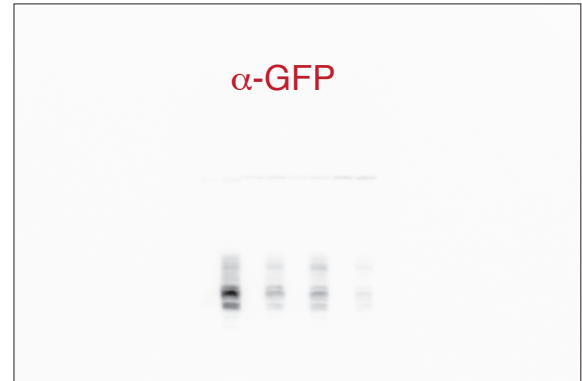

$\alpha$ -eEF2

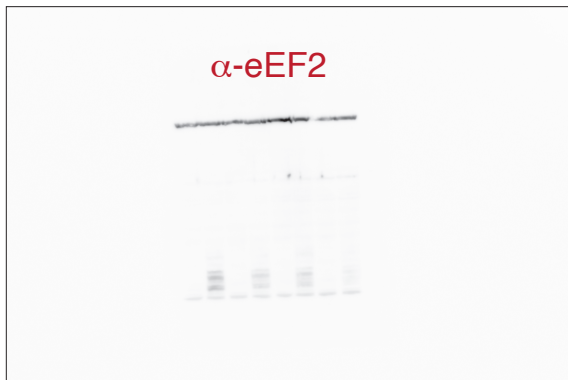

$\alpha$ -eEF2

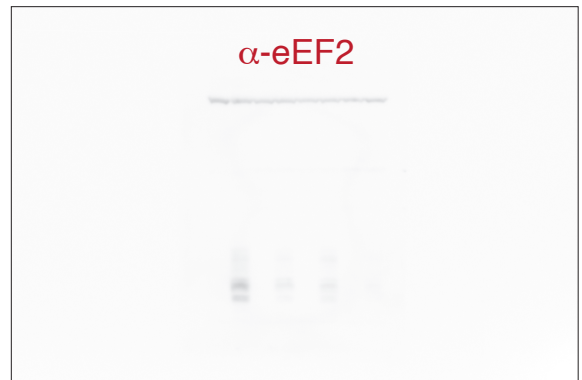

Supplement: Supplementary file 3 — Source data Fig. 1 [file 44318_2025_568_MOESM3_ESM.zip › Figure1/1B/Fig,1B_replicates.pdf]

Figure 1B

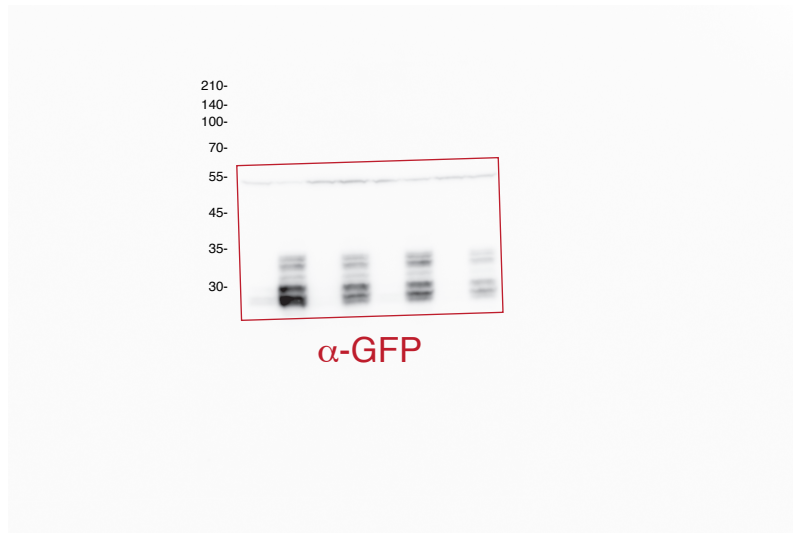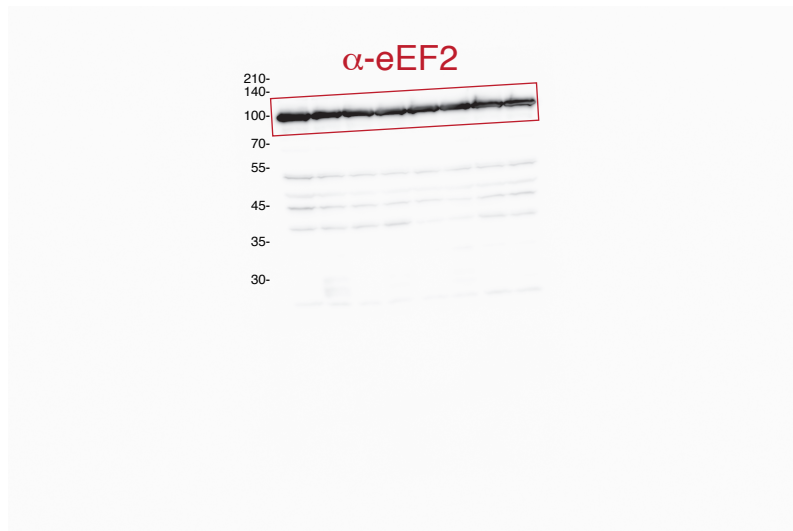

Supplement: Supplementary file 3 — Source data Fig. 1 [file 44318_2025_568_MOESM3_ESM.zip › Figure1/1B/Fig,1B.pdf]

Figure 1D

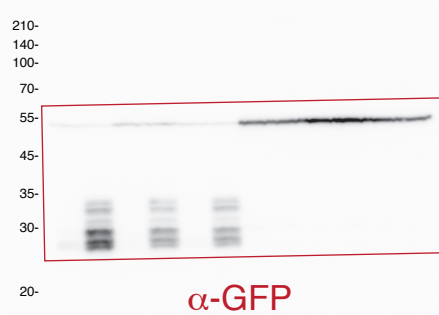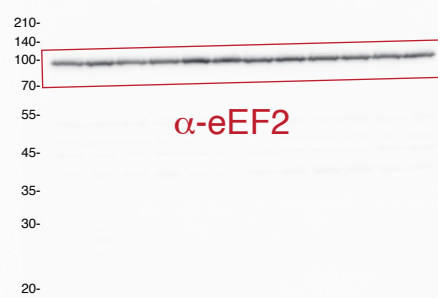

Supplement: Supplementary file 3 — Source data Fig. 1 [file 44318_2025_568_MOESM3_ESM.zip › Figure1/1D/Fig,1D.pdf]

Figure 1C

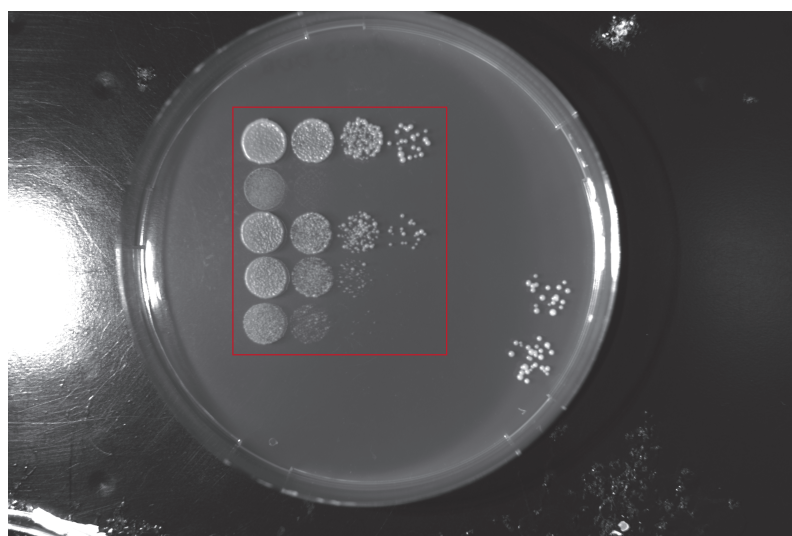

Supplement: Supplementary file 3 — Source data Fig. 1 [file 44318_2025_568_MOESM3_ESM.zip › Figure1/1C/Fig,1C.pdf]

Figure 2A

WT

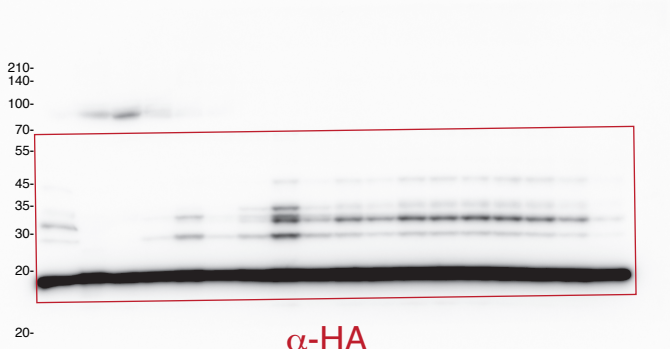

*ubp2* $\Delta$

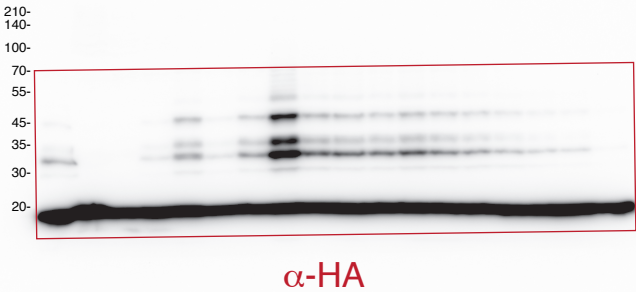

*ubp3* $\Delta$

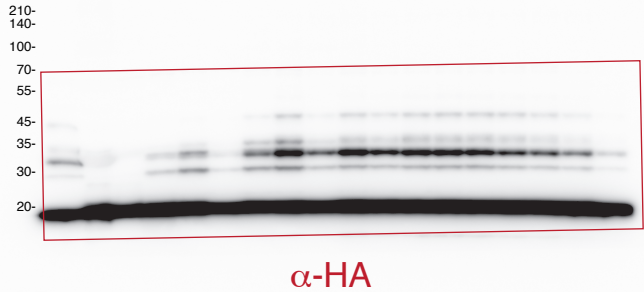

*ubp2* $\Delta$ *ubp3* $\Delta$

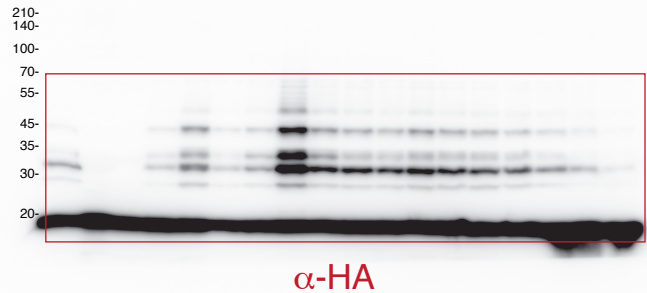

Supplement: Supplementary file 4 — Source data Fig. 2 [file 44318_2025_568_MOESM4_ESM.zip › Figure2/2A/Fig,2A.pdf]

Figure 2F

1.6 nM

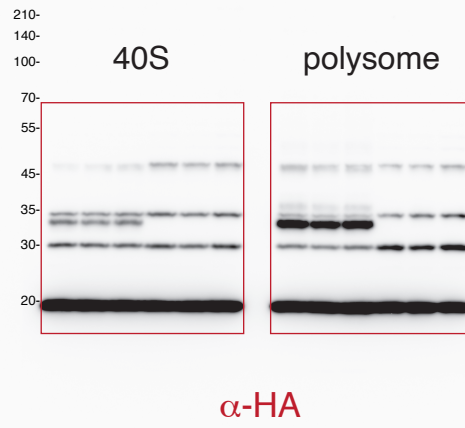

3.2 nM

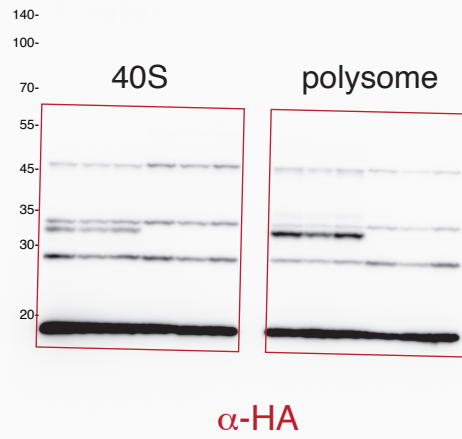

6.4 nM

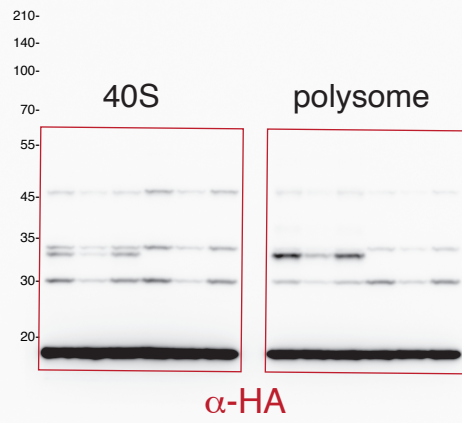

12.8 nM

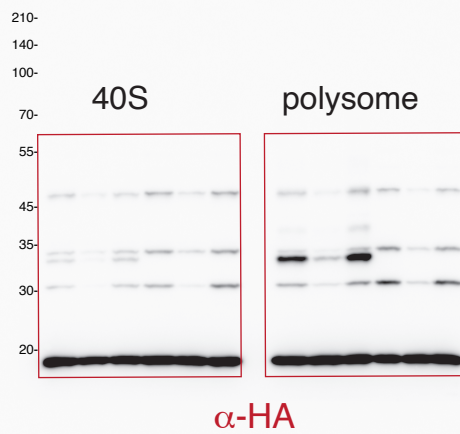

Supplement: Supplementary file 4 — Source data Fig. 2 [file 44318_2025_568_MOESM4_ESM.zip › Figure2/2F/Fig.2F.pdf]

Figure 2D

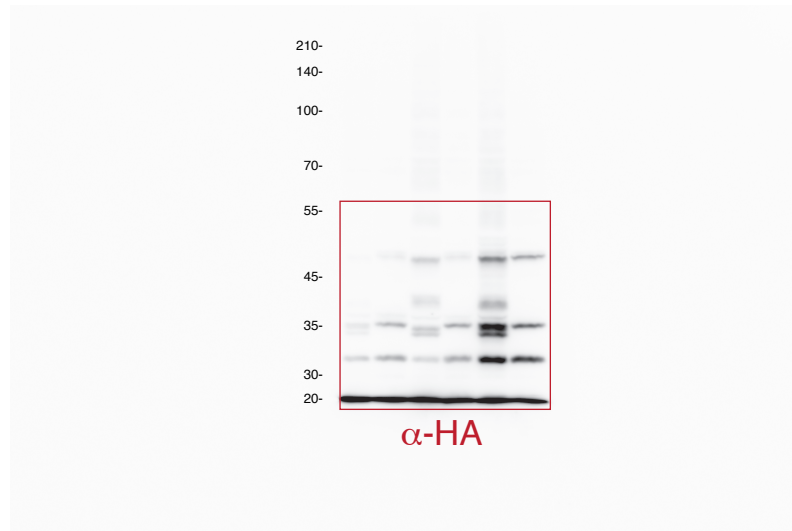

Supplement: Supplementary file 4 — Source data Fig. 2 [file 44318_2025_568_MOESM4_ESM.zip › Figure2/2D/Fig.2D.pdf]

Figure 2E

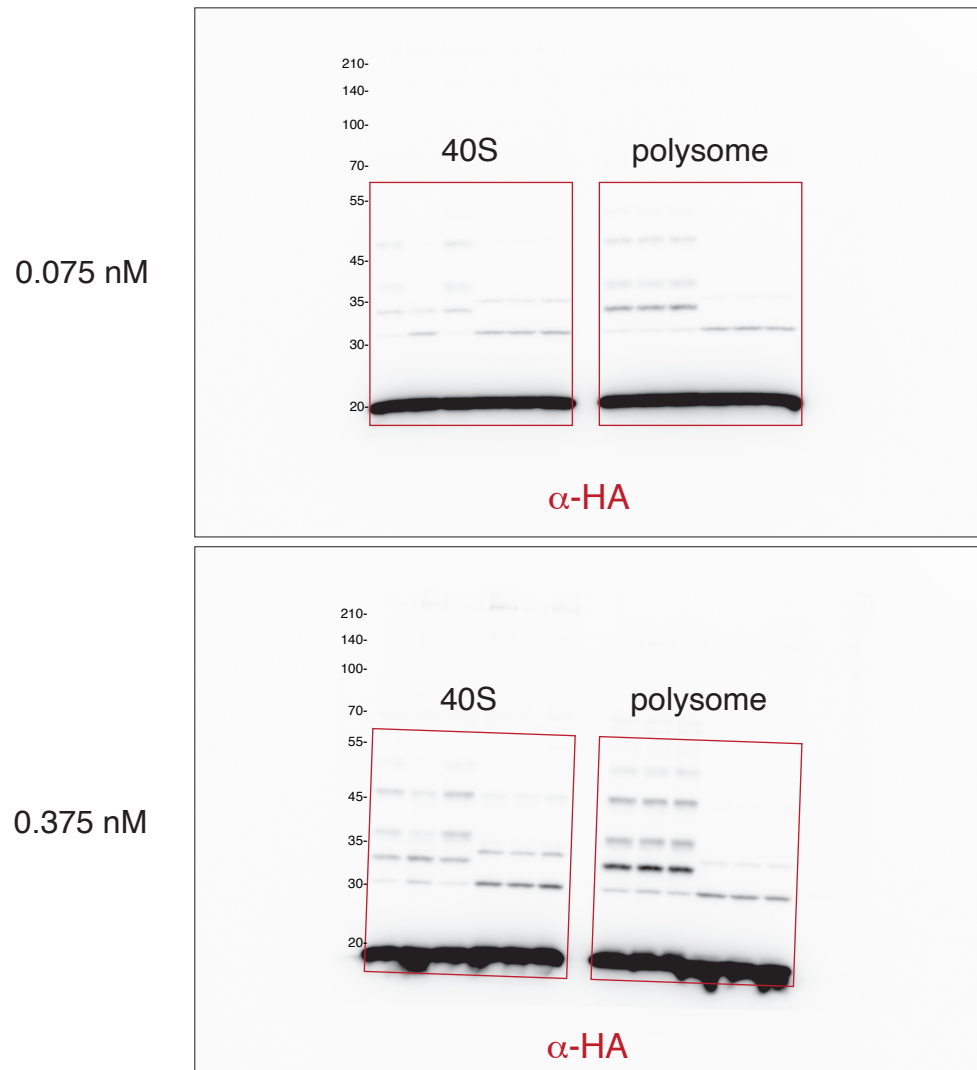

Supplement: Supplementary file 4 — Source data Fig. 2 [file 44318_2025_568_MOESM4_ESM.zip › Figure2/2E/Fig.2E.pdf]

Figure 3D

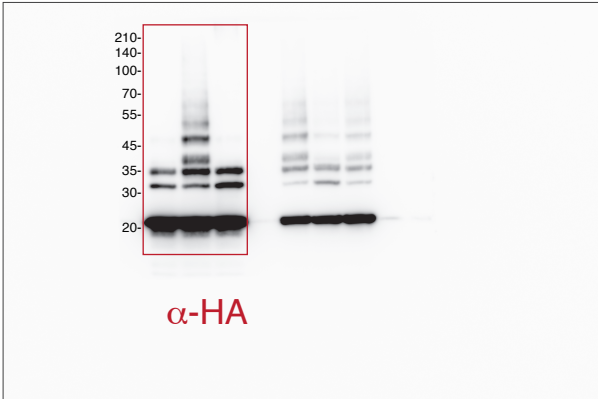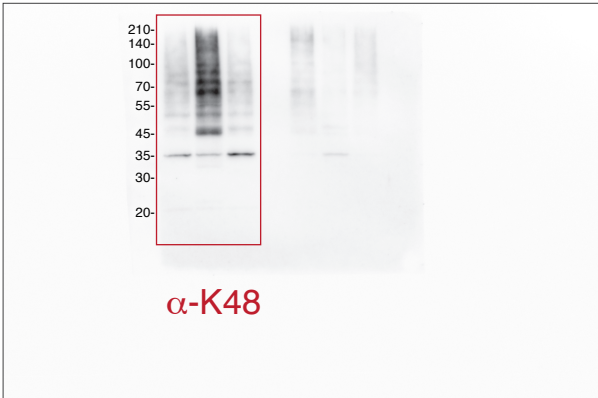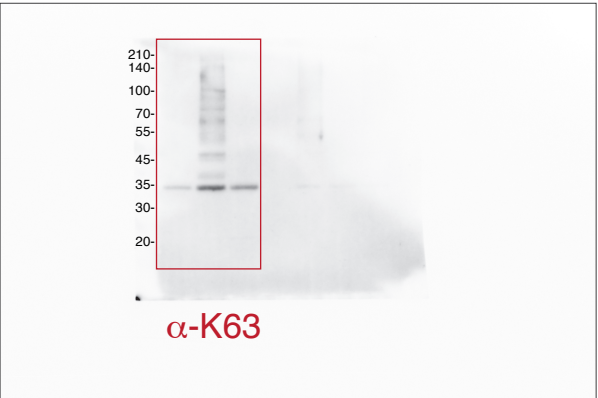

Supplement: Supplementary file 5 — Source data Fig. 3 [file 44318_2025_568_MOESM5_ESM.zip › Figure3/3D/Fig.3D.pdf]

Figure 4E

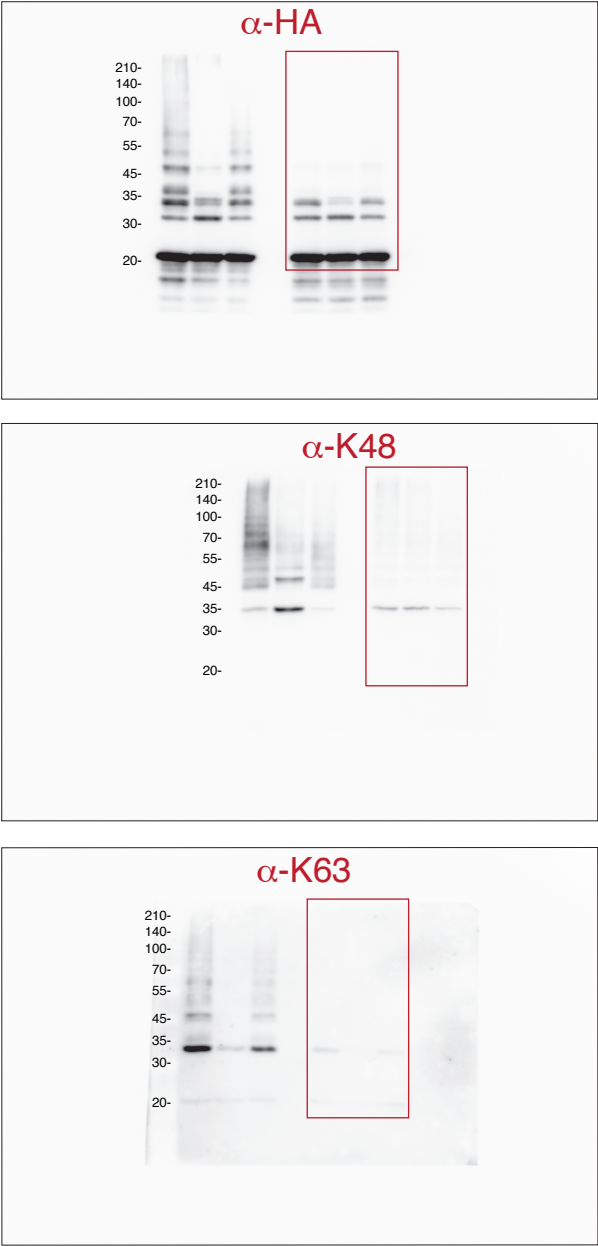

Supplement: Supplementary file 6 — Source data Fig. 4 [file 44318_2025_568_MOESM6_ESM.zip › Figure4/4E/Fig.4E.pdf]

Figure 4B

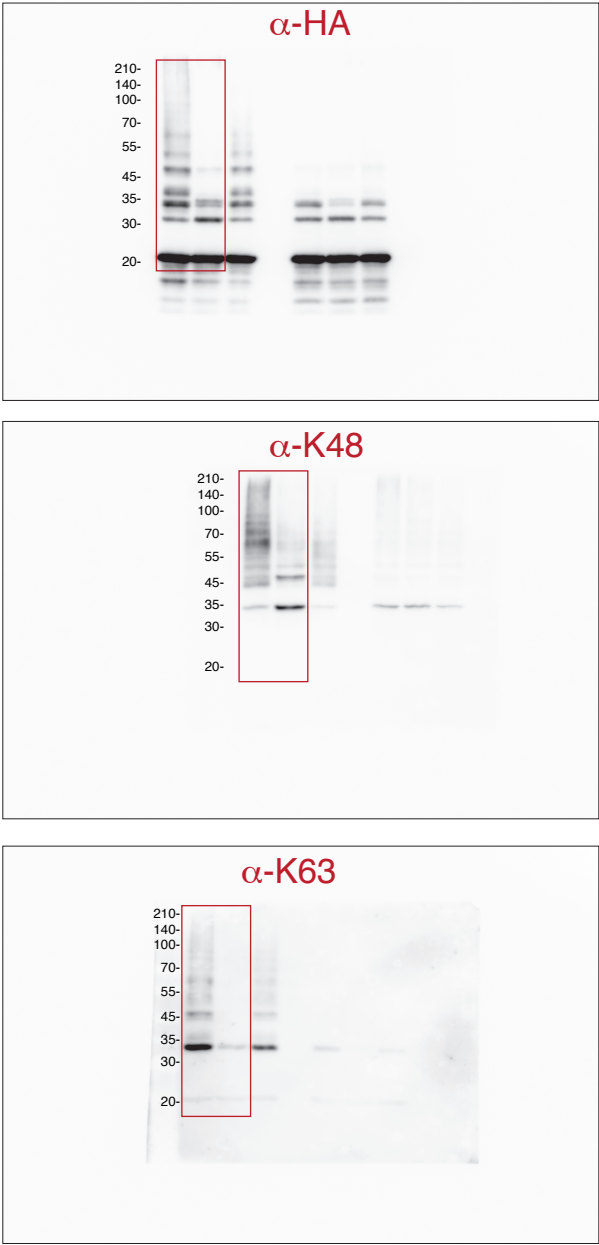

Supplement: Supplementary file 6 — Source data Fig. 4 [file 44318_2025_568_MOESM6_ESM.zip › Figure4/4B/Fig.4B.pdf]

Figure 4D

rep.2

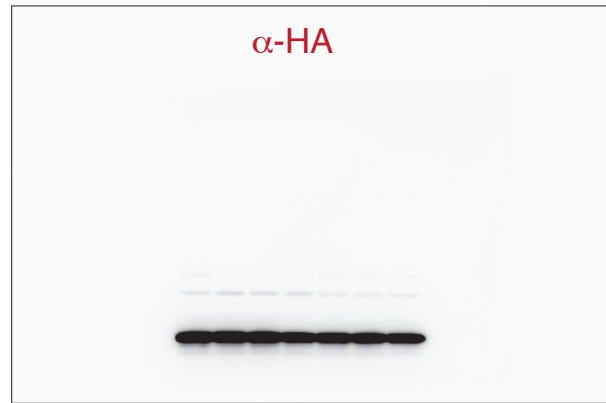

rep.3

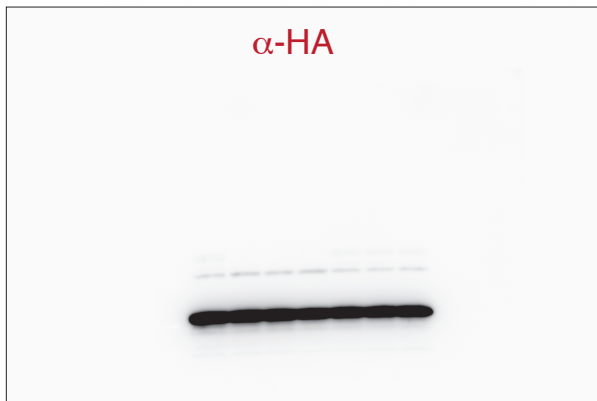

rep.4

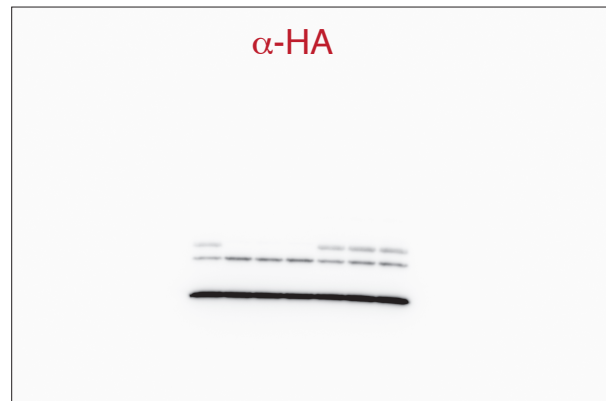

rep.5

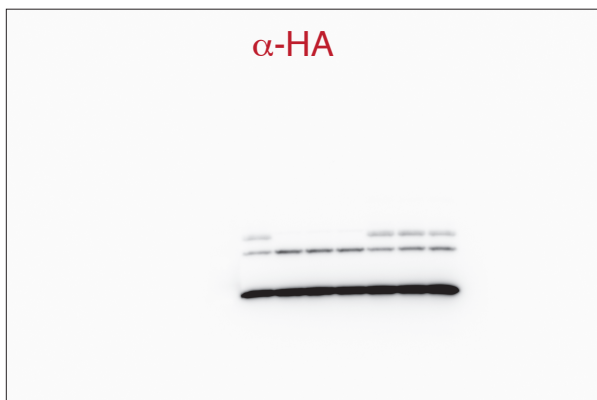

rep.6

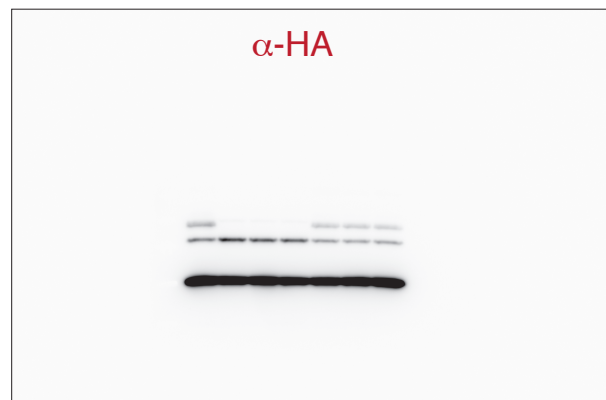

rep.7

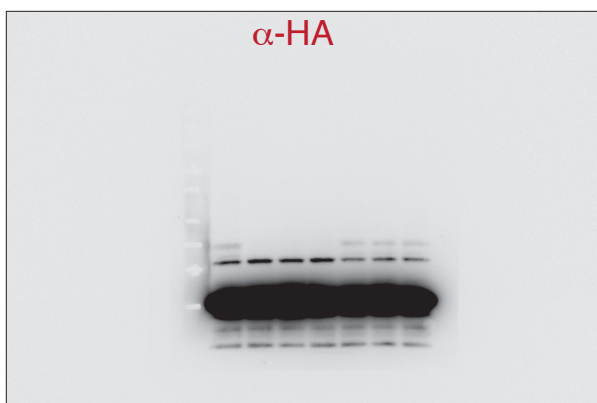

rep.8

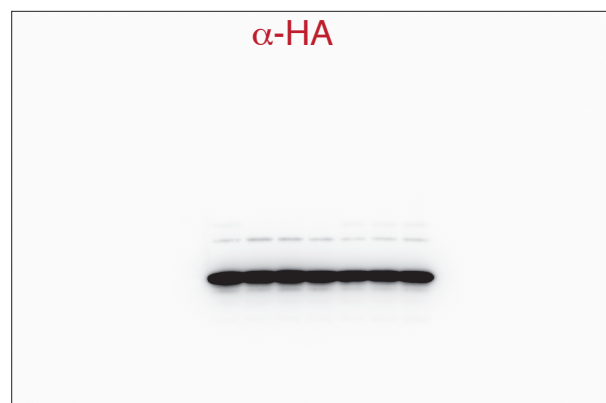

Supplement: Supplementary file 6 — Source data Fig. 4 [file 44318_2025_568_MOESM6_ESM.zip › Figure4/4D/Fig.4D_replicates.pdf]

Figure 4D

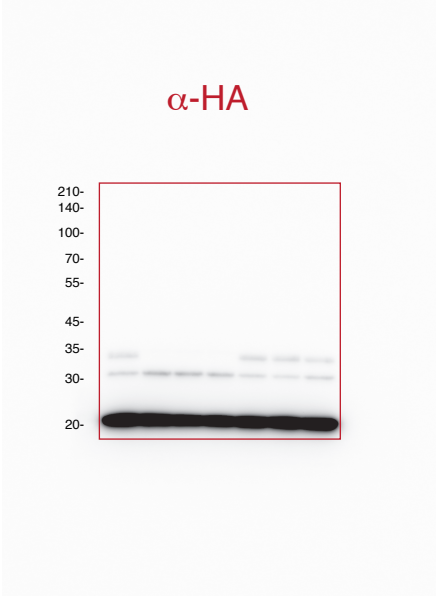

Supplement: Supplementary file 6 — Source data Fig. 4 [file 44318_2025_568_MOESM6_ESM.zip › Figure4/4D/Fig.4D.pdf]

Figure 4A

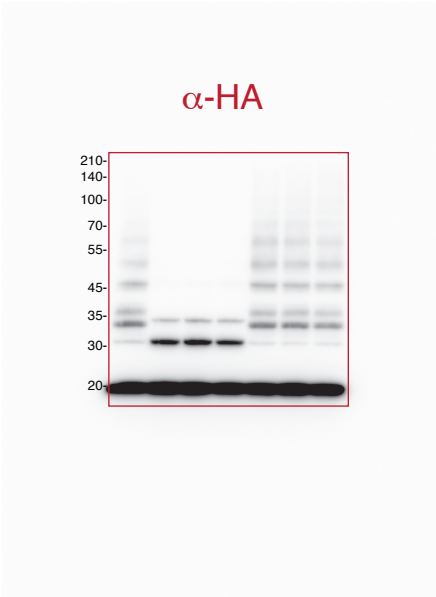

Supplement: Supplementary file 6 — Source data Fig. 4 [file 44318_2025_568_MOESM6_ESM.zip › Figure4/4A/Fig.4A.pdf]

Figure 4F

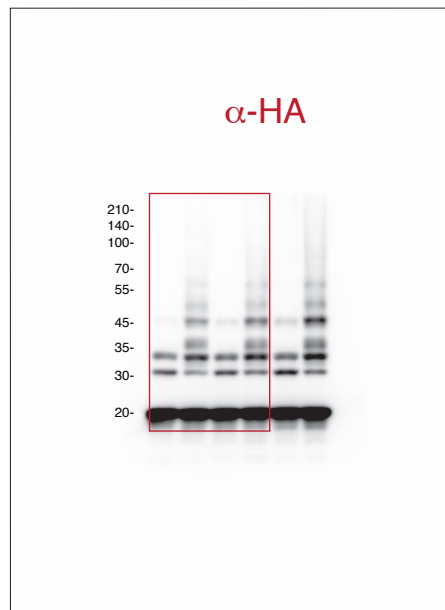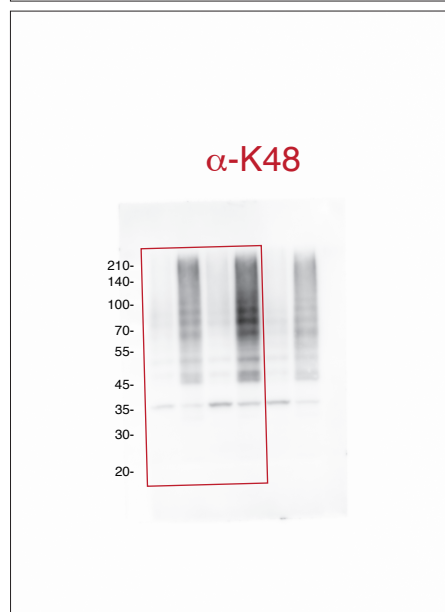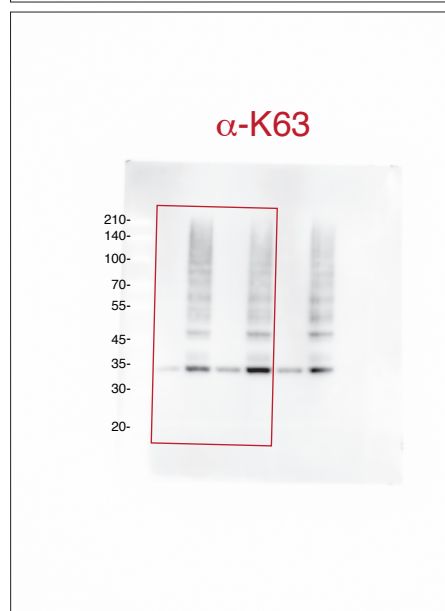

Supplement: Supplementary file 6 — Source data Fig. 4 [file 44318_2025_568_MOESM6_ESM.zip › Figure4/4F/Fig.4F.pdf]

Figure 5I

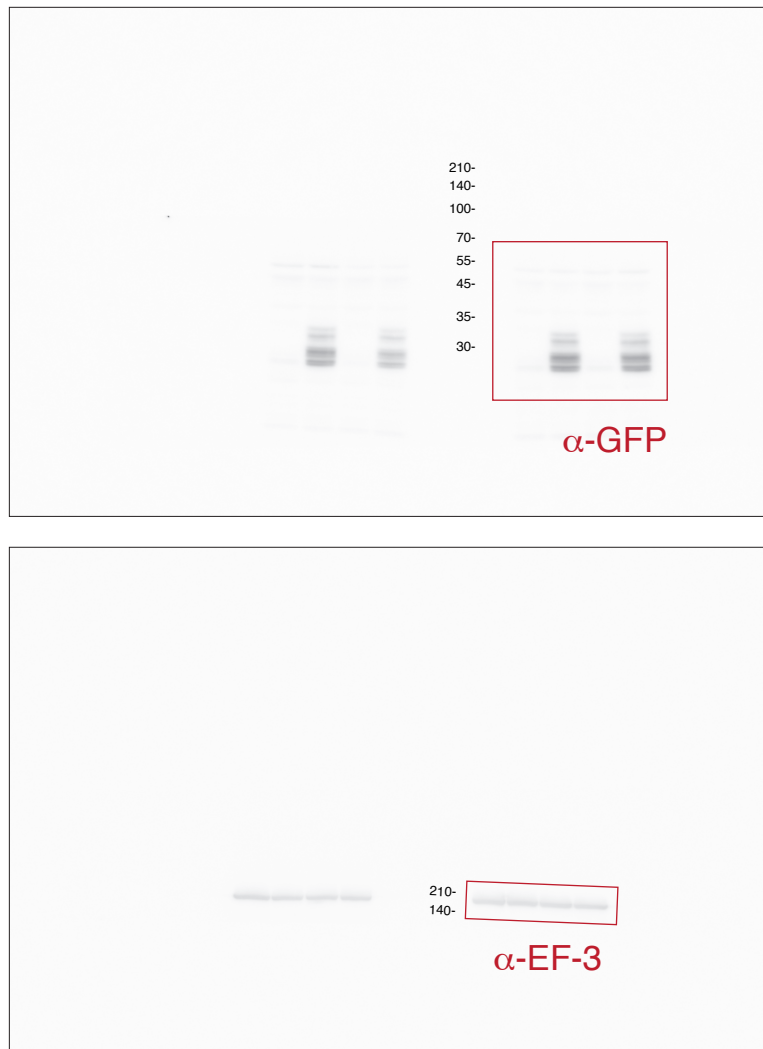

Supplement: Supplementary file 7 — Source data Fig. 5 [file 44318_2025_568_MOESM7_ESM.zip › Figure5/5I/Fig.5I.pdf]

Figure 5F

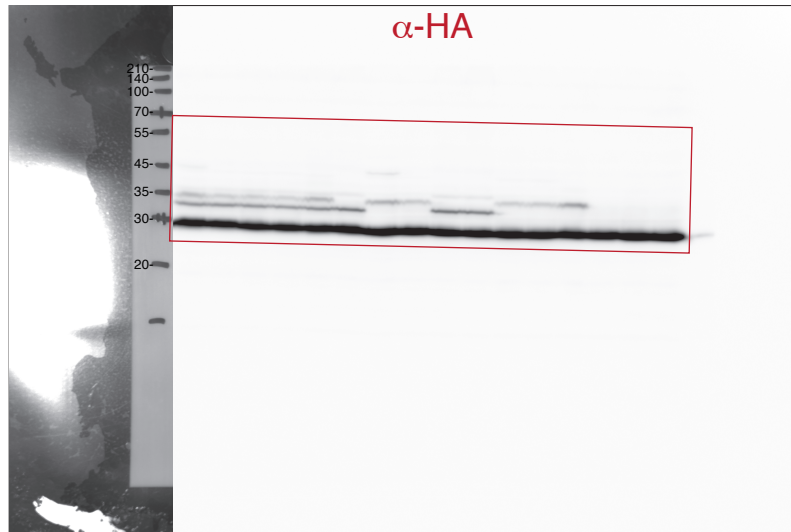

Supplement: Supplementary file 7 — Source data Fig. 5 [file 44318_2025_568_MOESM7_ESM.zip › Figure5/5F/Fig.5F.pdf]

Figure 5C

YPD

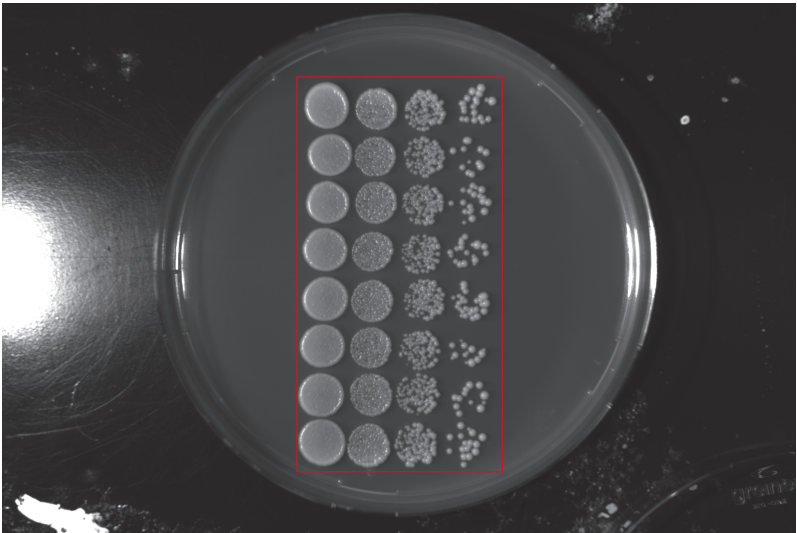

YPD  
+Anis.

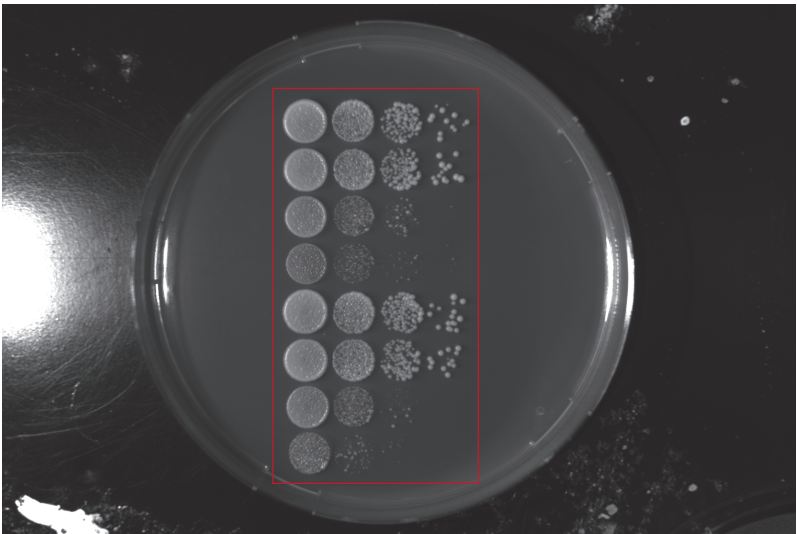

Supplement: Supplementary file 7 — Source data Fig. 5 [file 44318_2025_568_MOESM7_ESM.zip › Figure5/5C/Fig.5C.pdf]

Figure 5D

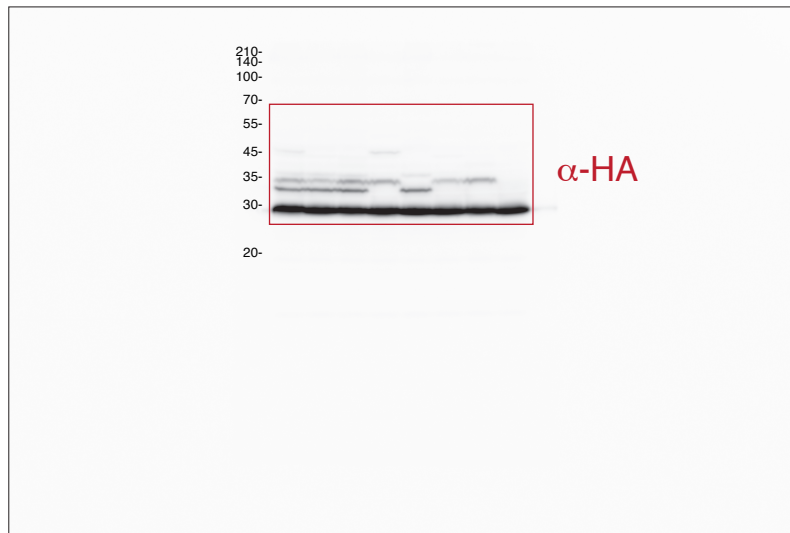

Supplement: Supplementary file 7 — Source data Fig. 5 [file 44318_2025_568_MOESM7_ESM.zip › Figure5/5D/Fig.5D.pdf]

Figure 5E

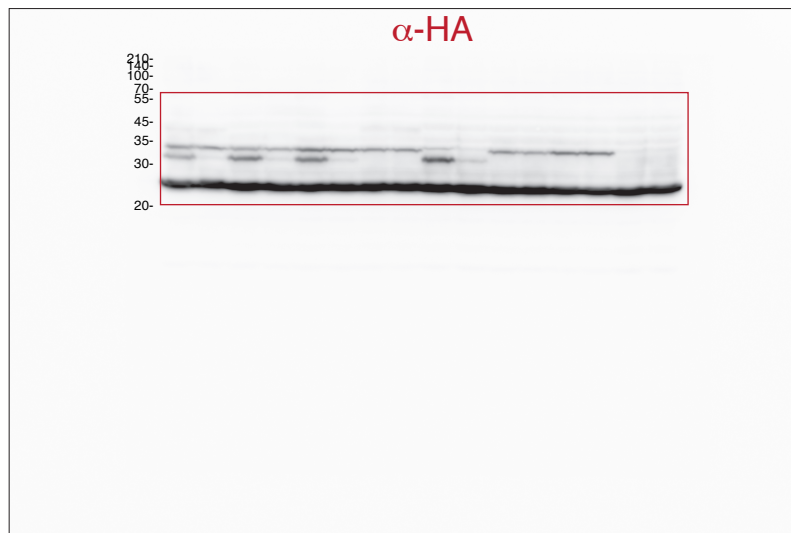

Supplement: Supplementary file 7 — Source data Fig. 5 [file 44318_2025_568_MOESM7_ESM.zip › Figure5/5E/Fig.5E.pdf]

Figure 5B

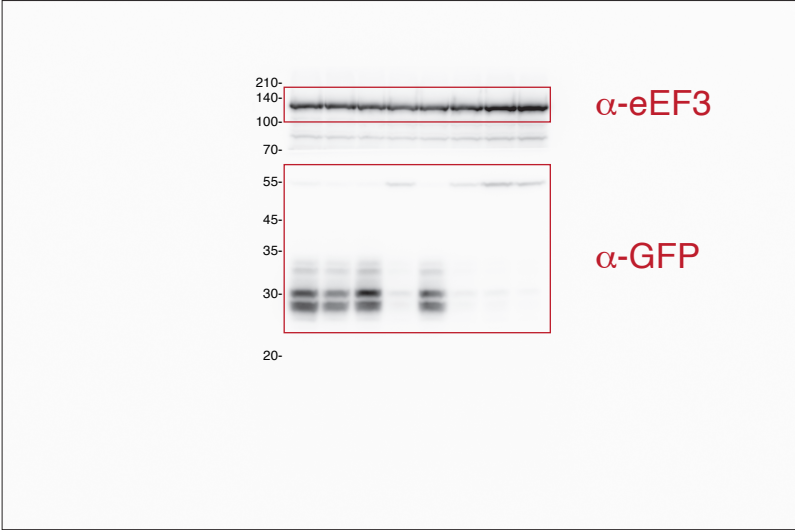

Supplement: Supplementary file 7 — Source data Fig. 5 [file 44318_2025_568_MOESM7_ESM.zip › Figure5/5B/Fig.5B.pdf]

Figure 6F

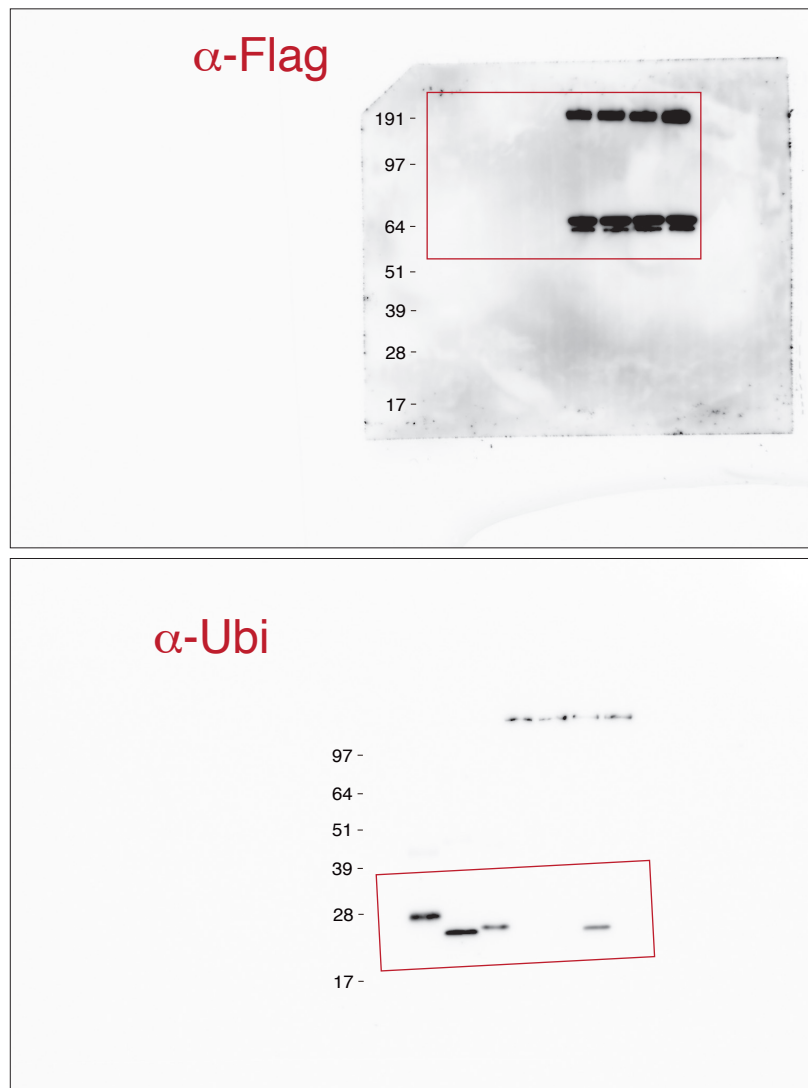

Supplement: Supplementary file 8 — Source data Fig. 6 [file 44318_2025_568_MOESM8_ESM.zip › Figure6/6F/Fig.6F.pdf]

Figure 6A

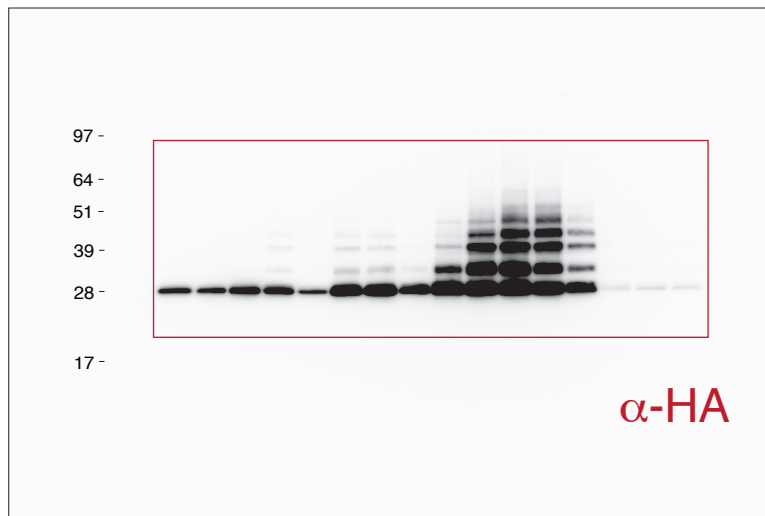

Supplement: Supplementary file 8 — Source data Fig. 6 [file 44318_2025_568_MOESM8_ESM.zip › Figure6/6A/Fig.6A.pdf]

Figure 6B

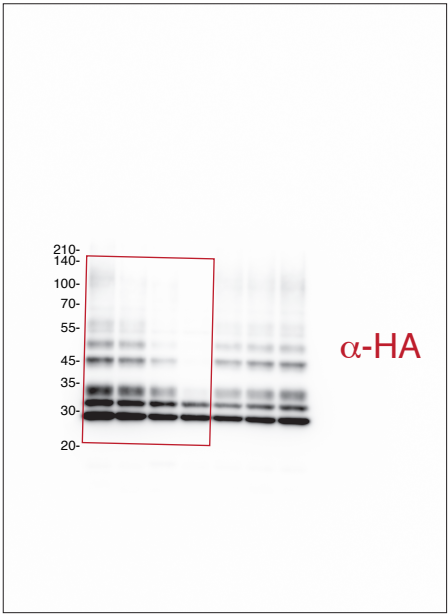

Supplement: Supplementary file 8 — Source data Fig. 6 [file 44318_2025_568_MOESM8_ESM.zip › Figure6/6B/Fig.6B.pdf]

Figure 6E

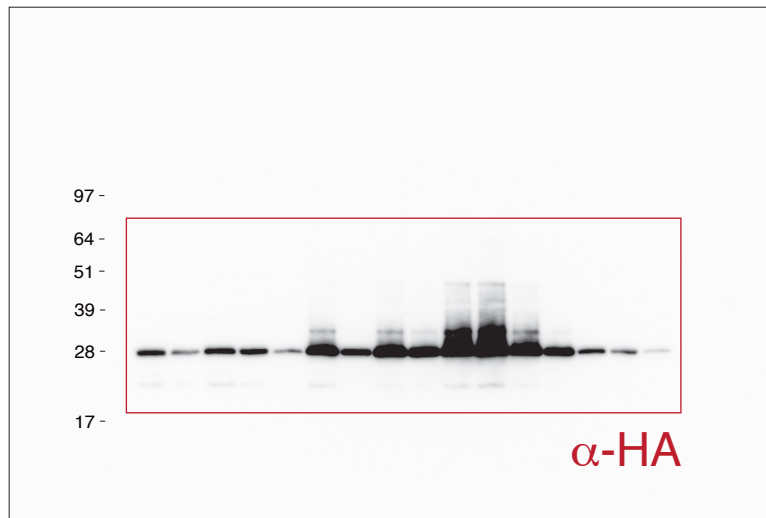

Supplement: Supplementary file 8 — Source data Fig. 6 [file 44318_2025_568_MOESM8_ESM.zip › Figure6/6E/Fig.6E.pdf]

Figure 6D

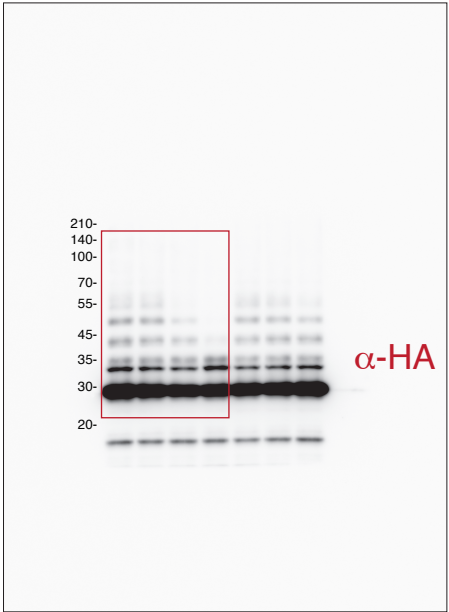

Supplement: Supplementary file 8 — Source data Fig. 6 [file 44318_2025_568_MOESM8_ESM.zip › Figure6/6D/Fig.6D.pdf]

Figure 6C

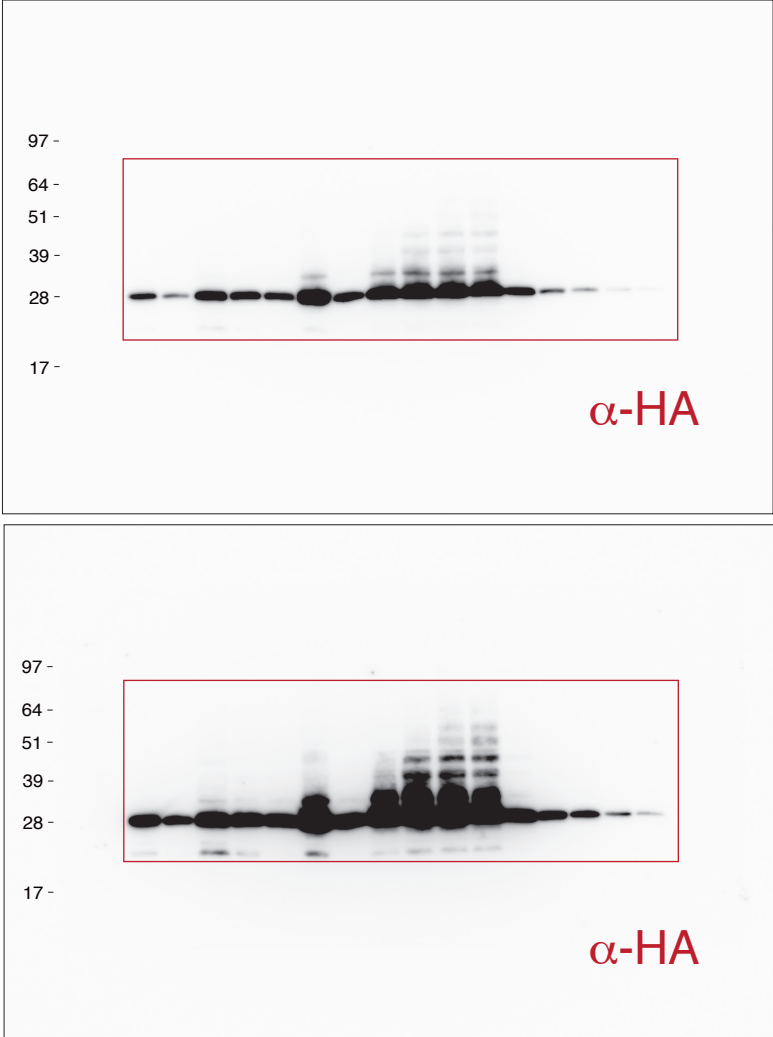

Supplement: Supplementary file 8 — Source data Fig. 6 [file 44318_2025_568_MOESM8_ESM.zip › Figure6/6C/Fig.6C.pdf]

Figure 7B

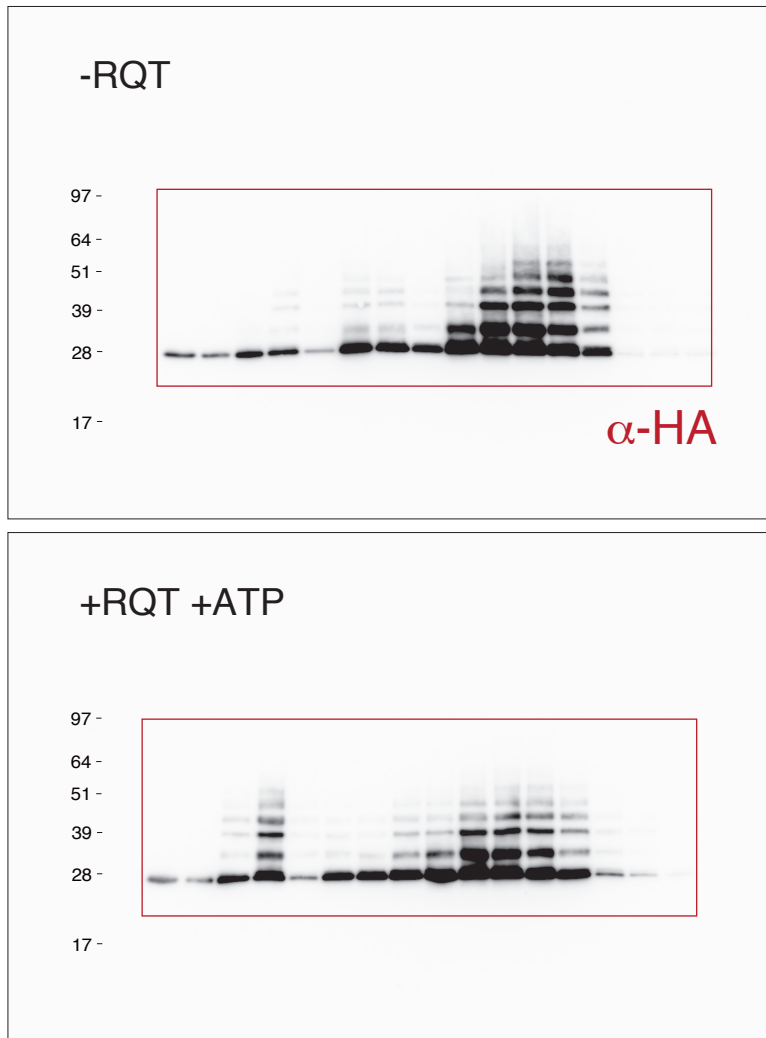

Supplement: Supplementary file 9 — Source data Fig. 7 [file 44318_2025_568_MOESM9_ESM.zip › Figure7/7B/Fig.7B.pdf]

Figure 7A

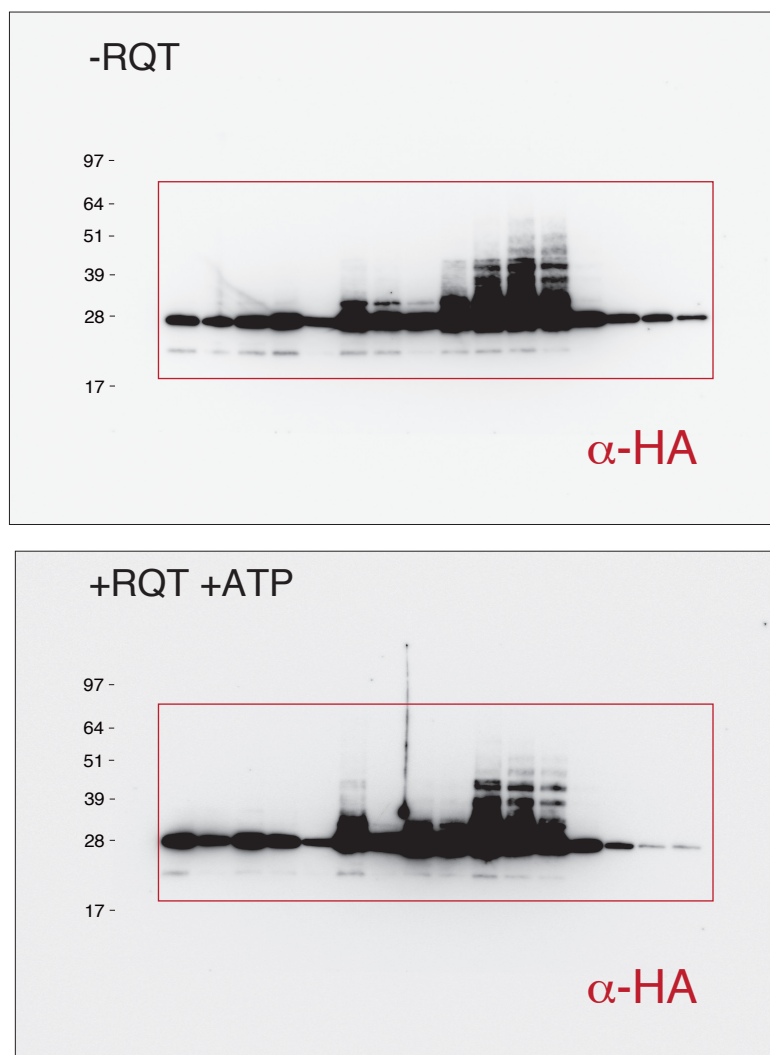

Supplement: Supplementary file 9 — Source data Fig. 7 [file 44318_2025_568_MOESM9_ESM.zip › Figure7/7A/Fig.7A.pdf]
