# Supplementary material for: Polyubiquitin architecture editing on collided ribosomes maintains persistent RQC activity
Source: EMBO J. 2025 Sep 16;44(21):6051–77. doi: 10.1038/s44318-025-00568-0 (PMC12583759; doi:10.1038/s44318-025-00568-0)

Figure 2A

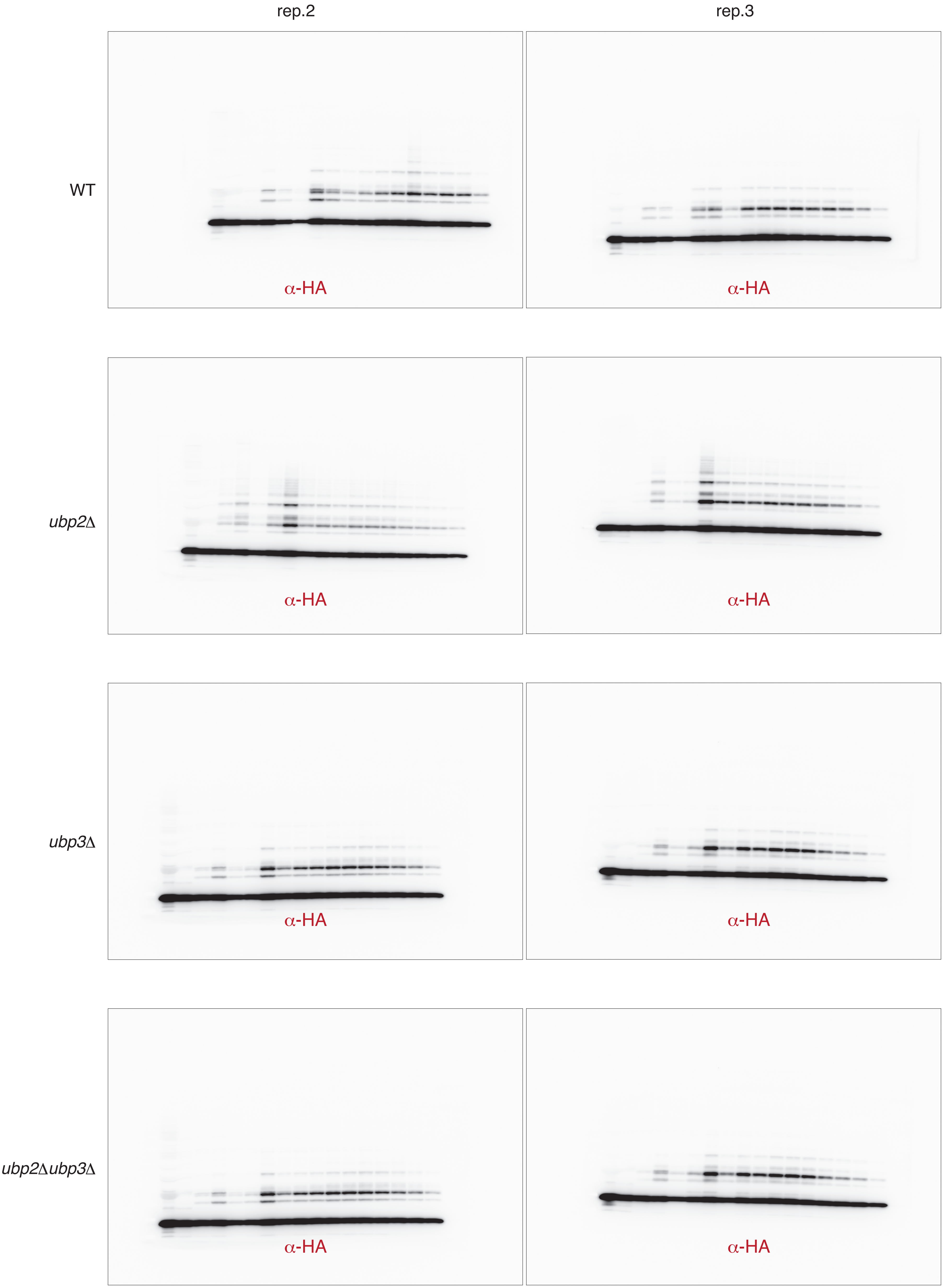

Supplement: Supplementary file 4 — Source data Fig. 2 [file 44318_2025_568_MOESM4_ESM.zip › Figure2/2A/Fig,2A_replicates.pdf]
