# Supplementary material for: Polyubiquitin architecture editing on collided ribosomes maintains persistent RQC activity
Source: EMBO J. 2025 Sep 16;44(21):6051–77. doi: 10.1038/s44318-025-00568-0 (PMC12583759; doi:10.1038/s44318-025-00568-0)
Supplement: Supplementary file 10 — Expanded View Figures [file 44318_2025_568_MOESM10_ESM.pdf]

Expanded View Figures

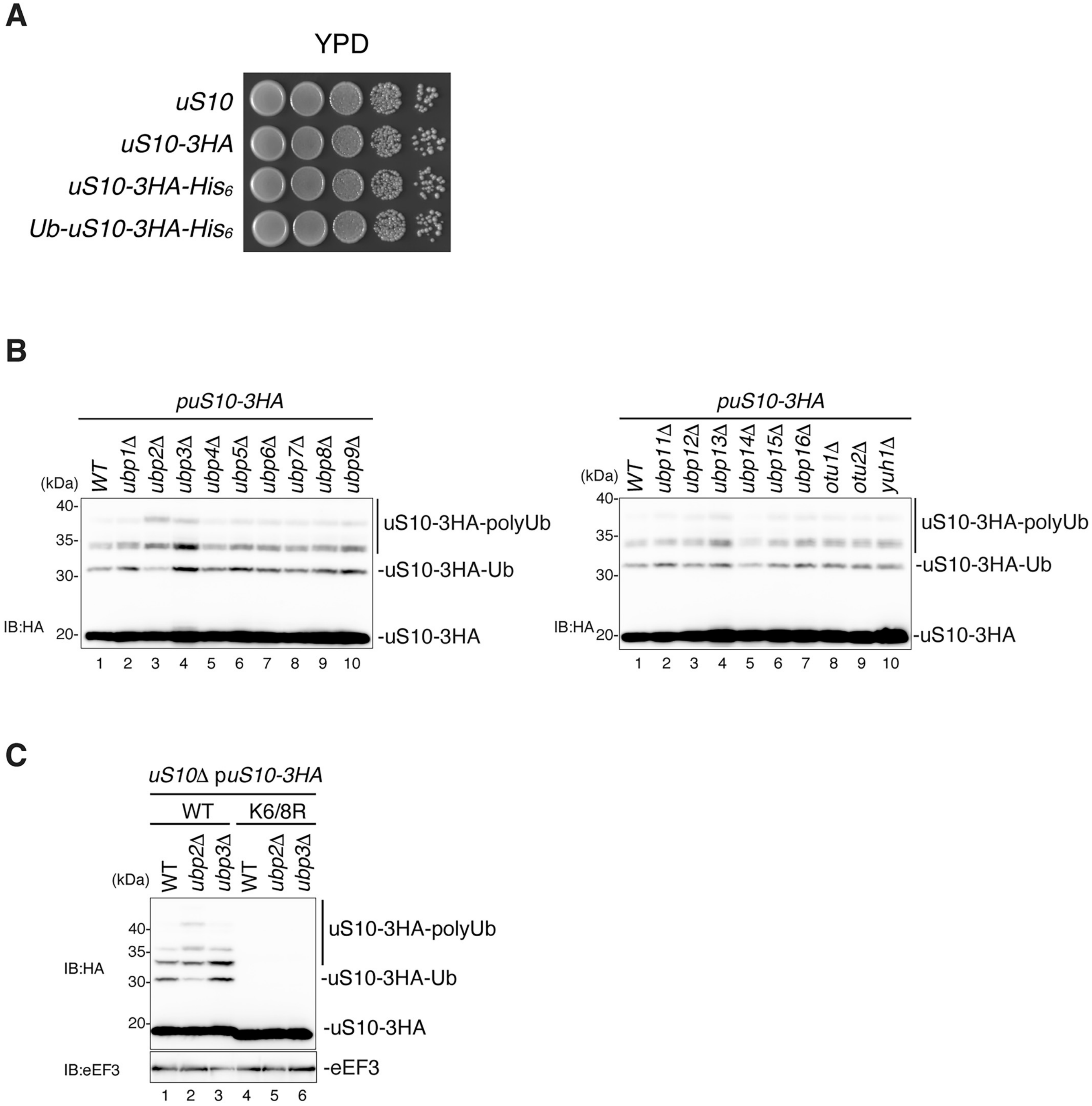

Figure EV1. Ubp2 and Ubp3 are involved in the deubiquitination of uS10.

(**A**) Spot assay of the indicated uS10 tagged or untagged strains. The indicating cells: *uS10Δ* expressing uS10, uS10-3HA, uS10-3HA-His$_6$, or Ub-uS10-3HA-His$_6$ from plasmids pKI124, pKI236, pST051, and pST158, respectively, diluted to $OD_{600} = 0.3$ and 10-fold serial dilutions were spotted and incubated at 30°C for two days. (**B**) Genetic screening to identify the deubiquitinating enzymes for uS10. Protein samples prepared from the indicated mutant cells: wild-type, *ubp1Δ, ubp2Δ, ubp3Δ, ubp4Δ, ubp5Δ, ubp6Δ, ubp7Δ, ubp8Δ, ubp9Δ, ubp11Δ, ubp12Δ, ubp13Δ, ubp14Δ, ubp15Δ, ubp16Δ, otu1Δ, otu2Δ, and yuh1Δ*, expressing uS10-3HA from plasmid pST001 were subjected to immunoblotting using an anti-HA antibody. Total proteins used for the immunoblotting were prepared by Cell lysis method. (**C**) The analysis of the ubiquitination of the uS10-K6/8 R mutant in the *ubp2Δ* and *ubp3Δ* strains. The ubiquitin level of uS10-3HA or uS10K6/8R-3HA derived from plasmid pKI236 or pKI237 in the *uS10Δ, uS10Δubp2Δ, uS10Δubp3Δ* mutants was detected with immunoblotting using an anti-HA antibody.

 © The Author(s)

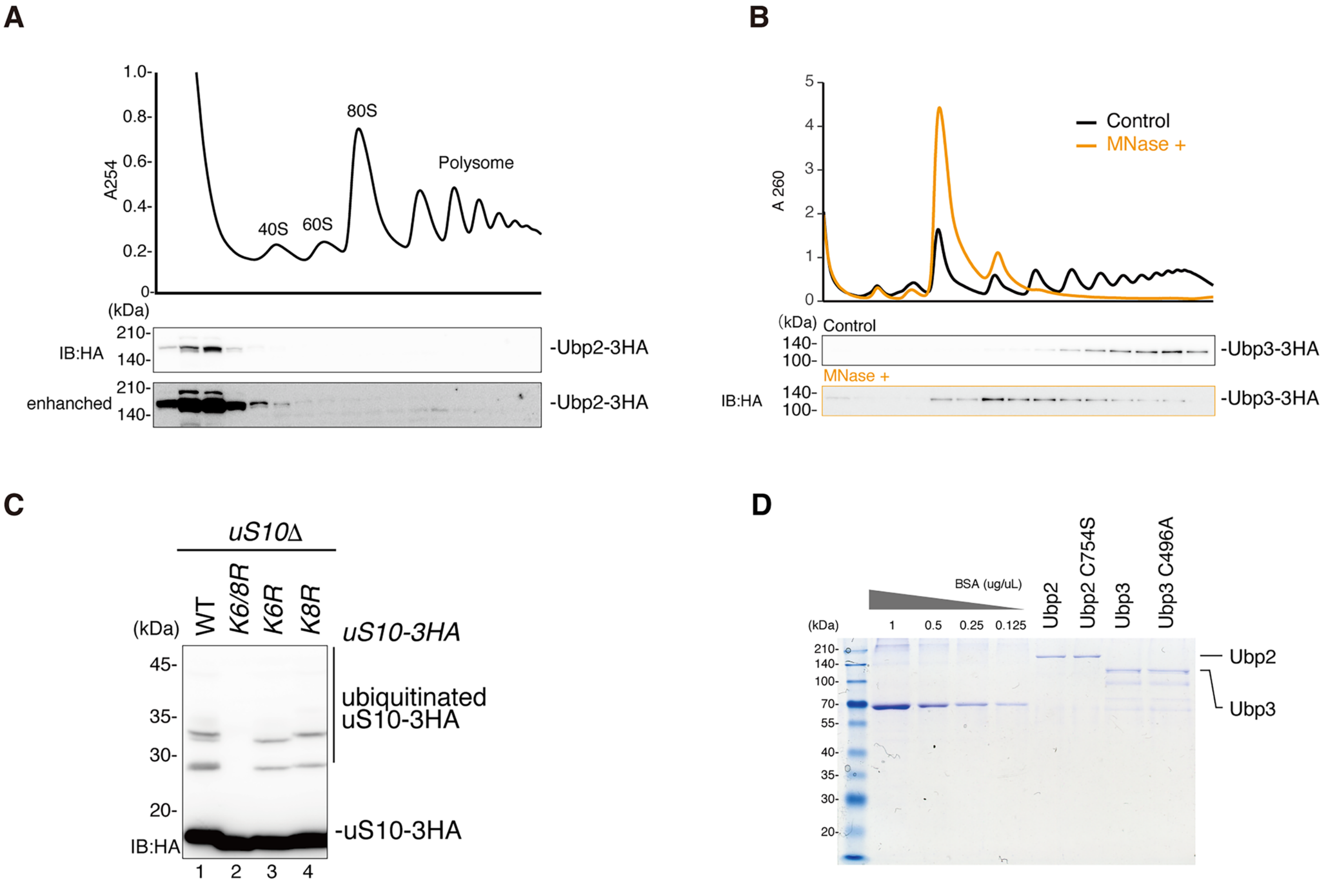

Figure EV2. Ubp2 and Ubp3 are associated with 40S subunit and polysome, respectively.

(**A**, **B**) The total lysates derived from 3 x HA genomic tagging Ubp2 (**A**) or Ubp3 (**B**) expressing cells were subjected to the sucrose density gradient and sedimented through by ultracentrifugation. For Ubp3, lysates were prepared either with or without micrococcal nuclease (MNase) treatment. Ubp2-3HA and Ubp3-3HA in each fraction were detected by immunoblotting using an anti-HA antibody. (**C**) The polyubiquitination of uS10 in K6R and K8R mutant of uS10. The 3 x HA-tagged uS10, uS10-K6/8 R, uS10-K6R or K8R was expressed from plasmids pKI237, pKI238, and pKI239, respectively, in the *uS10Δ* cells and detected the HA-tagged uS10 by immunoblotting using an anti-HA antibody. Total proteins used for immunoblotting were prepared by Cell lysis method. (**D**) CBB stain of purified Ubp2, Ubp2 C745S, Ubp3, Ubp3C496A.

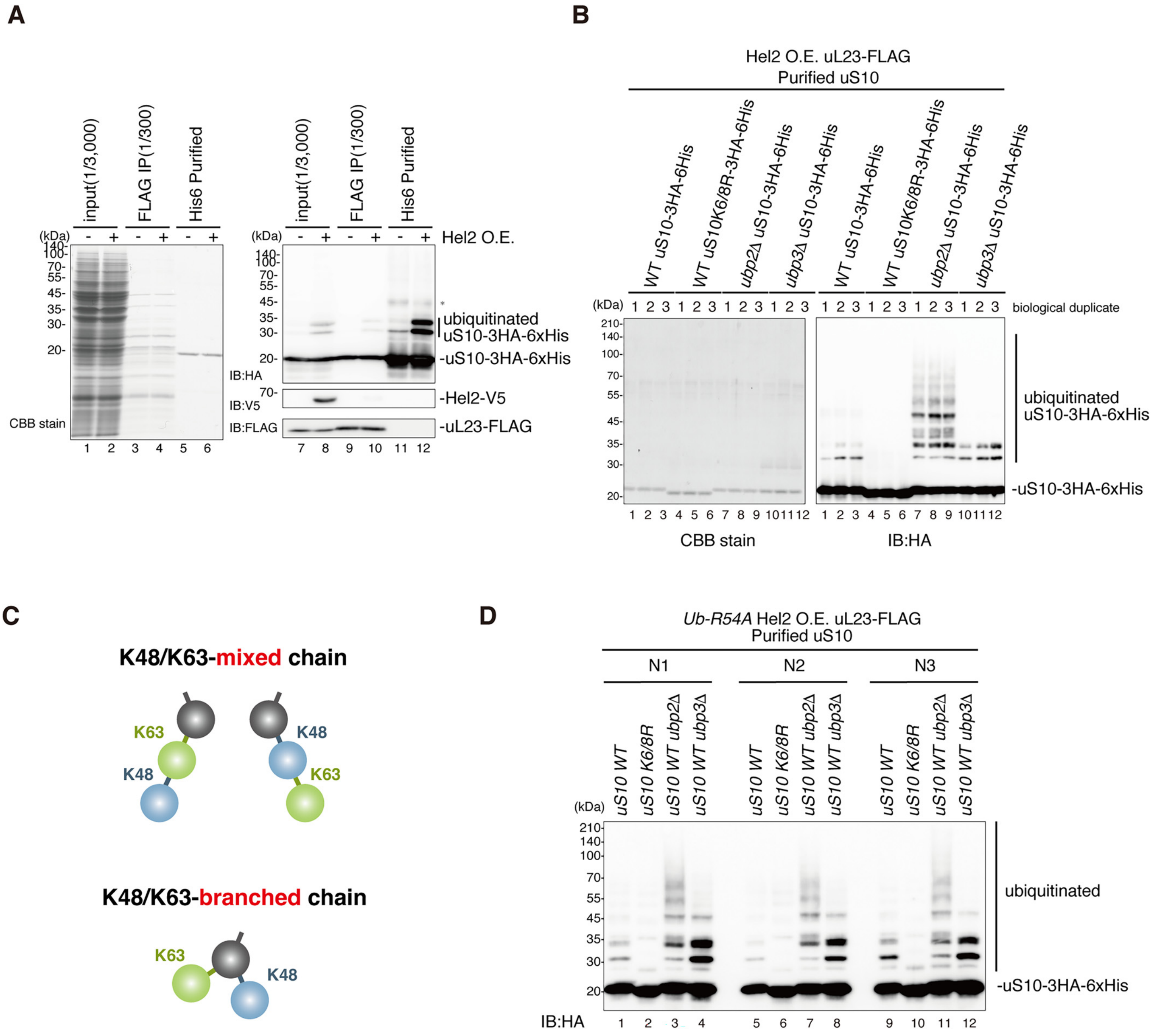

Figure EV3. The polyubiquitin chains formed on uS10 are mainly K63- and K48-linkage.

(**A**) The two-step purification of uS10-3HA-His$_6$. The uS10-3HA-His$_6$, uL23-FLAG, and Hel2-V5 were expressed from plasmids pST051, pKI191, and pST069 in *uS10Δ* cells. The ubiquitinated uS10-3HA-His$_6$ were purified by two-step affinity purification. The purified samples were stained with CBB (Left) or subjected to Immunoblotting using an anti-HA antibody, anti-V5, and anti-FLAG antibody (Right). (**B**) The sample preparation for Absolute quantification analysis of Ub chains. The uS10-3HA-His$_6$ or uS10-K6/8R-3HA-His$_6$ together with uL23-FLAG, and Hel2-V5 were expressed from plasmids pST051, pST052, pKI191, and pST069 in the following strains: *uS10Δ*, *uS10Δubp2Δ*, *uS10Δubp3Δ*. The ubiquitinated uS10-3HA-His$_6$ were purified by two-step affinity purification. The purified samples were stained with CBB (Left) or subjected to Immunoblotting using an anti-HA antibody (Right). (**C**) Schematic drawing of K48/K63 mixed or branched ubiquitin chain architectures. (**D**) The sample preparation for Absolute quantification analysis of Ub chains. The uS10-3HA-His$_6$ or uS10-K6/8R-3HA-His$_6$ together with uL23-FLAG, Hel2-V5, and Ub-R54A were expressed from plasmids pST051, pST052, pKI191, pST069, and pUB100-R54A in the following strains: *ubi1-4ΔuS10Δ*, *ubi1-4ΔuS10Δubp2Δ*, *ubi1-4ΔuS10Δubp3Δ*. The ubiquitinated uS10-3HA-His$_6$ were purified by two-step affinity purification. The purified samples were subjected to Immunoblotting using an anti-HA antibody.

 © The Author(s)

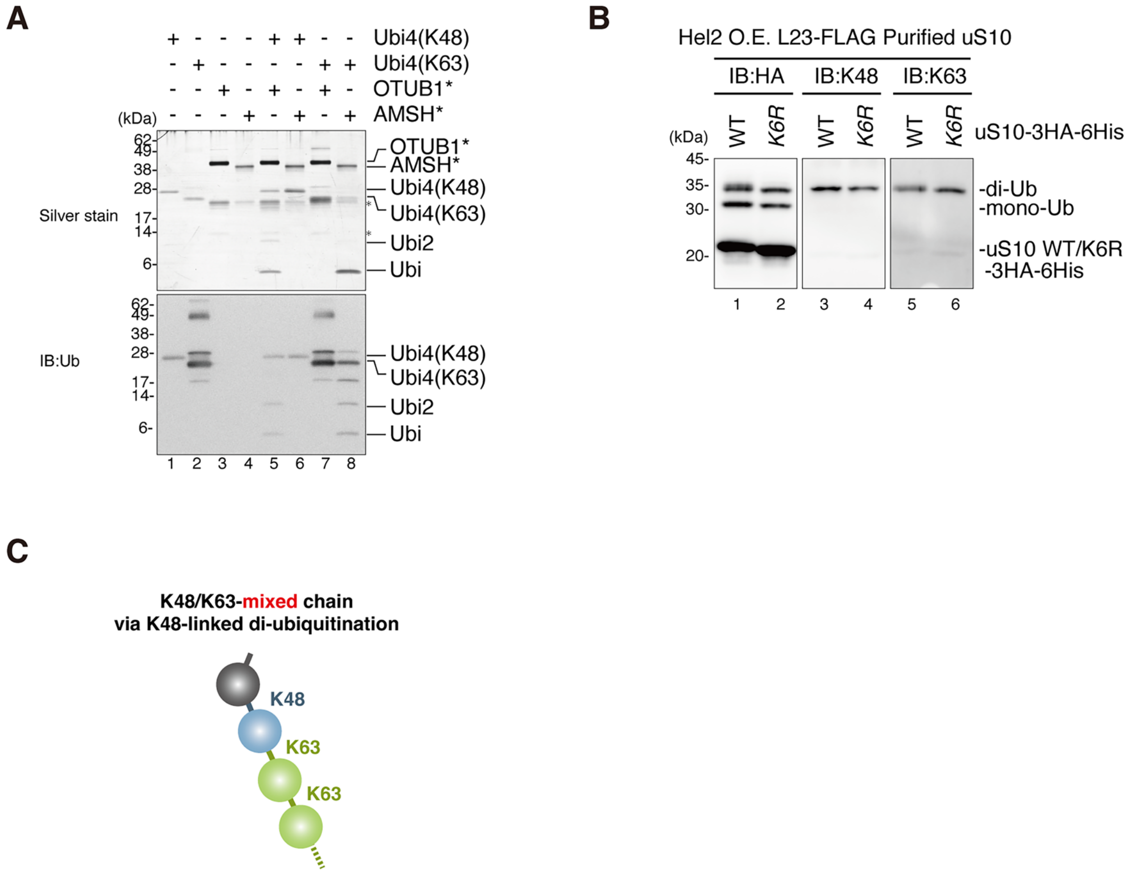

Figure EV4. The K48- and K63-linked di-ubiquitin chains are formed on uS10-K6R.

(**A**) K48-linked or K63-linked tetraubiquitin chains were reacted with AMSH* (K63-linkage-specific deubiquitinase) and OTUB1* (K48-linkage specific deubiquitinase). The protein samples were separated by 15% Nu-PAGE and detected by silver staining (top panel) or immunoblotting using an anti-ubiquitin antibody (bottom panel). (**B**) Both K48- and K63-linked polyubiquitin chain was formed on uS10. The uS10-WT-3HA-His$_6$ or uS10-K6R-3HA-His$_6$ together with uL23-FLAG and Hel2-V5 were expressed from plasmids pST051, pST137, pKI191, and pST069, respectively, in *uS10Δ* cells. The uS10-WT-3HA-His$_6$ or uS10-K6R-3HA-His$_6$ were purified by two-step affinity purification. Immunoblotting of purified samples using an anti-HA antibody, K48-linkage-specific anti-ubiquitin antibody, and K63-linkage-specific anti-ubiquitin antibody. (**C**) Schematic drawing of the architecture of K48/K63 mixed ubiquitin chain via K48-linked di-ubiquitination.

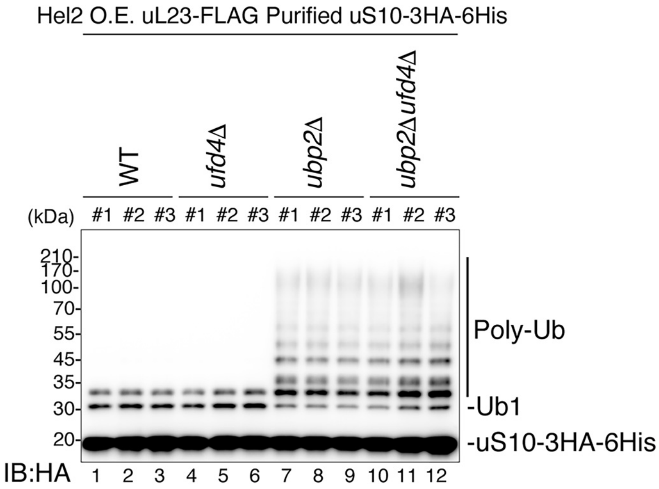

Figure EV5. The sample preparation for Absolute quantification analysis of Ub chains in the *ufd4*Δ mutant.

The uS10-3HA-His_6, uL23-FLAG, and Hel2-V5 were expressed from plasmids pST051, pKI191, and pST069 in the following strains: *uS10Δ*, *uS10Δufd4Δ*, *uS10Δubp2Δ*, *uS10Δufd4Δ*. The ubiquitinated uS10-3HA-His_6 were purified by two-step affinity purification. The purified samples were subjected to Immunoblotting using an anti-HA antibody.

 © The Author(s)

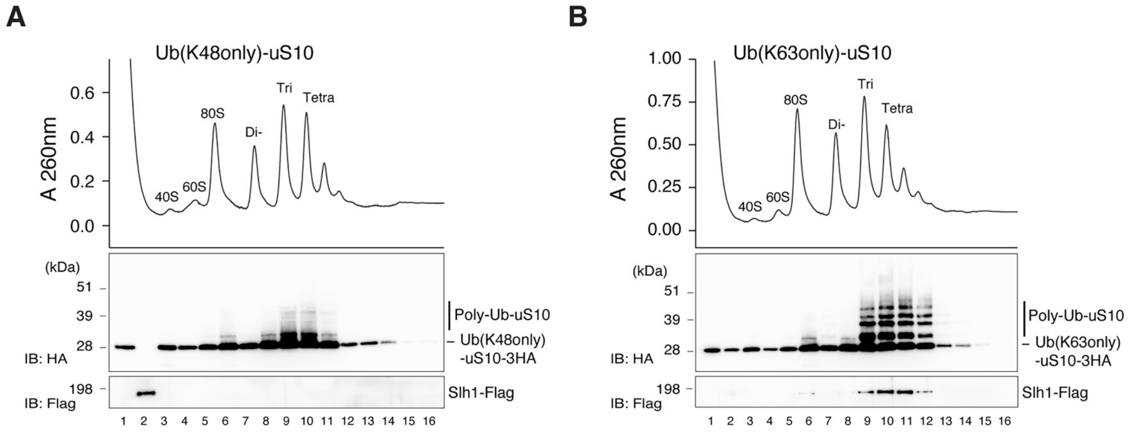

Figure EV6. RQT complex is associated with the K63-linked ubiquitinated colliding ribosomes but not with K48-K63-mixed polyubiquitinated colliding ribosomes.

The purified RNCs from the in vitro translation (IVT) reaction of the His-SDD1 model mRNA using the IVT extract prepared from Ub-K48only-uS10 mutant strain (**A**): *uS10Δski2Δ* expressing Ub-K48only-uS10-3HA from plasmid pST321, or Ub-K63only-uS10 mutant strain (**B**): *uS10Δski2Δ* expressing Ub-K63only-uS10-3HA from plasmid pST320, were incubated with the RQT complex in the absence of ATP and then separated by sucrose density gradient centrifugation. The ribosome abundance was detected by UV absorbance at 260 nm. HA-tagged uS10 and Flag-tagged Slh1, the component of the RQT complex, in each fraction, were detected by immunoblotting using anti-HA and anti-Flag antibodies, respectively.

© The Author(s)
